# Gene-lifestyle interactions in the genomics of human complex traits

**DOI:** 10.1038/s41431-022-01045-6

**Published:** 2022-03-22

**Authors:** Vincent Laville, Timothy Majarian, Yun J. Sung, Karen Schwander, Mary F. Feitosa, Daniel I. Chasman, Amy R. Bentley, Charles N. Rotimi, L. Adrienne Cupples, Paul S. de Vries, Michael R. Brown, Alanna C. Morrison, Aldi T. Kraja, Mike Province, C. Charles Gu, W. James Gauderman, the CHARGE Gene-Lifestyle Interactions Working Group, Vincent Laville, Timothy Majarian, Yun J. Sung, Karen Schwander, Mary F. Feitosa, Daniel I. Chasman, Amy R. Bentley, Charles N. Rotimi, L. Adrienne Cupples, Paul S. de Vries, Michael R. Brown, Alanna C. Morrison, Aldi T. Kraja, Mike Province, C. Charles Gu, W. James Gauderman, D. C. Rao, Alisa K. Manning, Hugues Aschard, D. C. Rao, Alisa K. Manning, Hugues Aschard

**Affiliations:** 1 Department of Computational Biology, Institut Pasteur, Université de Paris, F-75015 Paris, France; 2 Metabolism Program, Broad Institute of MIT and Harvard, Cambridge, MA 02142 USA; 3 Division of Biostatistics, Washington University, St. Louis, MO 63110 USA; 4 Division of Statistical Genomics, Department of Genetics, Washington University School of Medicine, St. Louis, MO 63108-221 USA; 5 Division of Preventive Medicine, Department of Medicine, Brigham and Women’s Hospital, Boston, MA 02215 USA; 6 Center for Research on Genomics and Global Health, National Human Genome Research Institute, National Institutes of Health, Bethesda, MD 20892 USA; 7 Department of Biostatistics, Boston University School of Public Health, Boston, MA 2118 USA; 8 Framingham Heart Study, National Heart, Lung, and Blood Institute, National Institutes of Health, Bethesda, MD 20982 USA; 9 Human Genetics Center, Department of Epidemiology, Human Genetics, and Environmental Sciences, School of Public Health, The University of Texas Health Science Center at Houston, Houston, TX 77030 USA; 10 Division of Biostatistics, Department of Population and Public Health Sciences, University of Southern California, Los Angeles, CA 90032 USA; 11 Clinical and Translational Epidemiology Unit, Massachusetts General Hospital, Boston, MA 02114 USA; 12 Department of Medicine, Harvard Medical School, Boston, MA 02115 USA; 13 Program in Genetic Epidemiology and Statistical Genetics, Harvard T.H. Chan School of Public Health, Boston, MA 02115 USA; 14 Department of Computational Biology, Institut Pasteur, Université de Paris, F-75015 Paris, France; 15 Metabolism Program, Broad Institute of MIT and Harvard, Cambridge, MA 02142 USA; 16 Division of Biostatistics, Washington University, St. Louis, MO 63110 USA; 17 Division of Statistical Genomics, Department of Genetics, Washington University School of Medicine, St. Louis, MO 63108-221 USA; 18 Division of Preventive Medicine, Department of Medicine, Brigham and Women’s Hospital, Boston, MA 02215 USA; 19 Center for Research on Genomics and Global Health, National Human Genome Research Institute, National Institutes of Health, Bethesda, MD 20892 USA; 20 Department of Biostatistics, Boston University School of Public Health, Boston, MA 2118 USA; 21 Framingham Heart Study, National Heart, Lung, and Blood Institute, National Institutes of Health, Bethesda, MD 20982 USA; 22 Human Genetics Center, Department of Epidemiology, Human Genetics, and Environmental Sciences, School of Public Health, The University of Texas Health Science Center at Houston, Houston, TX 77030 USA; 23 Division of Biostatistics, Department of Population and Public Health Sciences, University of Southern California, Los Angeles, CA 90032 USA; 24 Clinical and Translational Epidemiology Unit, Massachusetts General Hospital, Boston, MA 02114 USA; 25 Program in Genetic Epidemiology and Statistical Genetics, Harvard T.H. Chan School of Public Health, Boston, MA 02115 USA

**Keywords:** Genetics research, High-throughput screening, Medical genomics

## Abstract

The role and biological significance of gene-environment interactions in human traits and diseases remain poorly understood. To address these questions, the *CHARGE Gene-Lifestyle Interactions Working Group* conducted series of genome-wide interaction studies (GWIS) involving up to 610,475 individuals across four ancestries for three lipids and four blood pressure traits, while accounting for interaction effects with drinking and smoking exposures. Here we used GWIS summary statistics from these studies to decipher potential differences in genetic associations and G×E interactions across phenotype-exposure-ancestry combinations, and to derive insights on the potential mechanistic underlying G×E through in-silico functional analyses. Our analyses show first that interaction effects likely contribute to the commonly reported ancestry-specific genetic effect in complex traits, and second, that some phenotype-exposures pairs are more likely to benefit from a greater detection power when accounting for interactions. It also highlighted modest correlation between marginal and interaction effects, providing material for future methodological development and biological discussions. We also estimated contributions to phenotypic variance, including in particular the genetic heritability conditional on the exposure, and heritability partitioned across a range of functional annotations and cell types. In these analyses, we found multiple instances of potential heterogeneity of functional partitions between exposed and unexposed individuals, providing new evidence for likely exposure-specific genetic pathways. Finally, along this work, we identified potential biases in methods used to jointly meta-analyze genetic and interaction effects. We performed simulations to characterize these limitations and to provide the community with guidelines for future G×E studies.

## Introduction

The precise role of gene-environment interactions (G×E) in complex human traits and disease traits remains unclear. Although genome-wide G×E studies have been conducted for many phenotypes, the number of identified G×E is very small relative to the large number of genetic variants identified in traditional genome-wide association studies (GWAS). A number of issues related to the identification of G×E have been well described in the literature [1–3], including in particular very low power [4]. As a result, the required sample size needed to detect G×E is substantially larger than for marginal genetic effect (i.e., genetic effect estimated from a model not accounting for G×E). Moreover, few studies have explored potential differences in G×E across ancestry, assessed the contribution of G×E to the variance of human phenotypes, or explored enrichment of G×E for specific functional mechanisms.

The Gene-Lifestyle Interactions Working Group [5] within the Cohorts for Heart and Aging Research in Genetic Epidemiology (CHARGE) is an international initiative that has the potential to address some of these challenges. It is a large-scale, multi-ancestry consortium that aims at systematically evaluating genome-wide gene-lifestyle interactions on cardiovascular disease-related traits using genotypic data from up to 610,475 individuals. The consortium published a series of genome-wide single nucleotide polymorphism (SNP) by smoking and drinking interaction screenings focusing on four blood pressure phenotypes: diastolic blood pressure (DBP), systolic blood pressure (SBP), pulse pressure (PP), mean arterial pressure (MAP), and three lipid levels: triglycerides (TG), high-density lipoprotein cholesterol (HDL), and low-density lipoprotein cholesterol (LDL). For each pair of a phenotype and an exposure, a genome-wide interaction studies (GWIS) using the 1 degree of freedom (df) test for G×E interaction and the 2df joint test of main genetic effect (*i.e*. the estimate of genetic effect from the interaction model) and G×E interaction effects [6] has been conducted. The results from these analyses have been published in five papers: SNP-by-alcohol interaction [7] and SNP-by-smoking interaction [8, 9] on blood pressure, and SNP-by-alcohol interaction [10] and SNP-by-smoking interaction on lipids [11].

Here we first synthesize the GWIS results for all phenotype-exposure combinations. We highlight the importance of our large-scale initiative, providing evidence that interacting variants might differ by genetic ancestry, and show that accounting for G×E can help to discover new loci, especially for certain phenotype-exposure pairs. We then performed a series of analyses comparing interaction effects against marginal genetic effects derived from both our studies and from previous GWAS. Contrary to a commonly assumed hypothesis [12], we found only modest correlation between interaction effect and marginal effect, highlighting additional challenges for future G×E interactions studies. Estimated variance explained by main and interaction effect for the outcomes under study also showed that in general, interactions explain a very small amount of phenotypic variance on top of the marginal genetic effect for these traits. However, these limitations were balanced by stratified heritability analyses. Partitioning the genetic variance in exposed and unexposed individuals separately, using both functional and cell-type annotations, we observed differential enrichment patterns between the two groups in multiple instances. This suggests G×E might still play an important role in these phenotypes, with some exposures potentially triggering new molecular mechanisms or reducing the contribution of pathways involved in unexposed individuals.

## Methods

### Data and processing

We considered four blood pressure phenotypes (DBP, SBP, PP, MAP), and three lipids levels (TG, HDL, LDL). Two binary smoking exposures, *current smoking* and *ever smoking*, were considered and measured similarly across all smoking GWIS. The *current smoking* variable was coded as 1 if the subject smoked regularly in past year and as 0 otherwise. *Ever smoking* status was coded as 1 if the subject smoked at least 100 cigarettes during his/her lifetime and 0 otherwise. For alcohol consumption, two binary variables were considered, referred further as *current drinking* and *drinking habit*. All studies conducted a two-stage approach. In stage 1 (referred to as *Discovery*), a standard GWIS was performed using up to 18 million genetic variants. In stage 2 (referred to as *Replication*), only a subset of variants with a *p* value for either 1df or the 2df test below a certain threshold (*P* < 10^−6^ or *P* < 10^−5^) at stage 1 were further considered. For each outcome exposure, we had access to complete meta-analysis summary statistics of both the discovery and the replication stages for four different ancestries (European, African, Asian, and Hispanic) after quality control filtering. To ensure a fair comparison, we re-processed all results for each outcome-exposure-ancestry combination using the same pipeline. More details are provided on the data and pre-processing are available in the supplementary notes and in the corresponding publications [7–11].

### Identification of independent signals and associated loci

We defined two levels of association when reporting genome-wide significant variants in the combined meta-analyses (*P* < 5 × 10^−8^): independent signal (lead SNPs after clumping) and associated locus (genetic regions of 1 Mb with at least one independent signal). *Independent signals* represent independent SNPs associated at genome-wide significance level. Independent signals were defined using the clumping framework from the PLINK software [13], using a linkage disequilibrium (LD) threshold of 0.2 and a maximum physical distance from the lead SNP (*i.e*., the most associated variant) of ±500 kb. The LD was derived using 1000 Genomes Project [14] individuals as a reference panel while accounting for ancestry. We used the EUR, AFR, combined EAS-SAS, and AMR samples as proxies for the individuals from European ancestry (EA), African ancestry (AA), Asian ancestry (ASA), and Hispanic ancestry (HA), respectively. For the trans-ancestry analyses, we built our reference panel by merging all those reference populations. *Associated loci* are genetic regions of 1 Mb or more harboring at least one genome-wide associated SNPs. To define associated loci, we first derived region 500 kb upstream and downstream of each and every independent signal (as defined above). All overlapping regions were then merged to form the loci. Further details are provided in the Supplementary Note

### Interaction effect conditional on marginal effect

We assessed potential enrichment for interactions effects for SNPs displaying marginal genetic association. To increase independence between our interaction effect GWIS and the marginal GWAS, we used summary statistics from previous studies on blood pressure traits [15–17] and lipid traits [18–21]. However, note that there might be a small overlap of samples between some of these previously published marginal GWAS and the 1df and 2df GWIS from the CHARGE consortium. For this analysis, we considered only individuals of EA, in order to maximize the sample size while limiting potential issues due to genetic heterogeneity, where the top variants might differ across populations. In practice we used the 1df interaction test from the combined analysis derived in European sample in CHARGE, and for external studies, we used only the GWAS conducted in European populations. Moreover, to avoid enrichment driven by a single locus, we performed a clumping of the previously published GWAS of marginal genetic effect with PLINK [13], so that all lead SNPs considered are independent from each other. We first derived the proportion of interaction effect nominally significant at type I error rate (alpha) threshold of 0.05 among bins of SNPs grouped based on their marginal association (i.e., we derive the proportion of SNPs with interaction *p* value below 0.05 and *p* value for marginal effect in bins [1, 0.1], [0.1, 0.01], [0.01, 0.001], etc). Note that the aforementioned clumping of SNPs avoids any biased enrichment due to pairwise SNP correlation. For the last bin, including only SNPs previously identified at genome-wide significance level (5 × 10^−8^) in marginal effect GWAS, we also performed three complementary association tests [4] to assess interaction effects that would have been missed by single SNP G×E interaction: an omnibus test, an unweighted genetic risk score (uGRS) test, and a weighted genetic risk score (wGRS) (see Supplementary Note).

### Variance explained and heritability

For each ancestry and each phenotype-exposure combination, we derived from the combined (stage 1 and stage 2) results the fraction of phenotypic variance explained by top SNPs was decomposed into main effects, interaction effects and those effects jointly using the R package *VarExp* [22]. The significance of the variance explained by interaction effects was derived using an approximation of the joint test of all interaction effects. For EA samples, we further assessed potential differences in heritability across exposure-specific strata using stage 1 genome-wide association results using the *LDSC* approach [23]. We used the pre-computed *LDscore* relative to EA samples provided with the software. When unavailable from the original studies, stratified results were derived from the interaction model using J2S [24]. For each exposure stratum, genetic heritability was further partitioned by both cell-type-specific and general annotations [25] using two distinct sets of annotations: *baseline* and *GenoSkyline*+. The significance of the annotation enrichment was assessed using a Bonferroni corrected significance threshold of *P* < 0.000277. Tissue-specific heritability was also derived following Finucane et al. [26]. Except when specified otherwise, enrichment analyses compared median enrichment between exposure strata. We avoided comparison of significance level here, which would be biased by differences in sample size. Additional details of these analyses are provided in the Supplementary Note.

## Results

### Overview

We investigated results from 28 GWIS on three lipid and four blood pressure phenotypes, each examining G×E interaction with two smoking and two alcohol exposures (Table 1). All outcome-exposure pairs were analyzed using a two-stage approach involving up to 610,475 individuals. In stage 1, a GWIS was performed in up to 29 cohorts with a total of up to 149,684 individuals from four ancestries: EA, AA, ASA, and HA. In stage 2, involving up to 71 additional cohorts including 460,791 individuals, also from multiple ancestries, studies focused on the replication of a subset of variants from stage 1. The total sample size (discovery and replication) varied substantially across the trait analyzed, with an average of 311 K for lipids and 457 K for blood pressure traits. Moreover, our analyses explored not only the 28 primary trans-ancestry GWIS, but also the 112 corresponding ancestry-specific GWIS. To ensure a fair comparison across all analyses, we re-processed all GWIS summary results using the same pipeline. Stage 1 quantile-quantile (QQ) plots for both the 1df and the 2df test are presented in Fig. S1, and frequency of the exposure are presented in Fig. S2 and Table S1. Finally, note that the primary association results from the original studies and our analyses are highly concordant, but minor differences might exist because of slight differences in the analysis pipeline.

**Table 1:** Summary of trans-ancestry GWIS results for 2df joint and 1df interaction tests.

Outcome		Exposure	# variants	Sample size^a^ (disc)	Sample size^a^ (rep)	# Loci (signals)^b^ 2df	# loci (signals)^b^ 1df
Lipids	HDL	Current drinking	7,505,310	127,252	231,043	111 (584)	0 (0)
		Drinking habits	6,848,811	118,899	217,468	109 (528)	0 (0)
		Current smoking	6,306,314	133,508	253,467	69 (335)	0 (0)
		Ever smoking	7,269,995	133,816	251,711	74 (370)	0 (0)
	LDL	Current drinking	7,448,913	118,654	171,142	92 (492)	0 (0)
		Drinking habits	6,834,699	111,093	155,280	78 (446)	0 (0)
		Current smoking	6,261,354	125,629	188,109	53 (251)	0 (0)
		Ever smoking	7,251,615	125,638	186,230	45 (163)	0 (0)
	TG	Current drinking	7,410,534	104,716	221,722	71 (413)	0 (0)
		Drinking habits	6,839,760	103,214	210,623	72 (365)	0 (0)
		Current smoking	7,122,377	111,900	241,140	52 (220)	0 (0)
		Ever smoking	8,438,564	111,909	238,972	49 (226)	0 (0)
Blood pressure	SBP	Current drinking	7,489,960	121,948	426,121	55 (106)	0 (0)
		Drinking habits	10,639,279	62,479	114,058	29 (47)	0 (0)
		Current smoking	6,849,695	127,730	474,475	66 (139)	0 (0)
		Ever smoking	7,928,860	127,733	458,034	68 (137)	0 (0)
	DBP	Current drinking	7,490,269	121,947	426,177	57 (101)	0 (0)
		Drinking habits	10,639,829	62,479	114,111	31 (42)	0 (0)
		Current Smoking	6,784,799	127,730	474,531	70 (138)	0 (0)
		Ever smoking	7,930,829	127,730	458,089	66 (136)	0 (0)
	MAP	Current drinking	7,489,903	121,947	426,112	48 (71)	0 (0)
		Drinking habits	10,639,231	62,479	113,287	32 (46)	0 (0)
		Current smoking	6,848,964	127,730	474,465	69 (144)	1 (1)
		Ever smoking	7,932,503	127,730	458,024	67 (137)	0 (0)
	PP	Current drinking	7,489,921	121,947	420,767	39 (67)	0 (0)
		Drinking habits	10,639,279	62,479	114,111	18 (27)	0 (0)
		Current smoking	7,934,402	127,730	473,514	54 (92)	0 (0)
		Ever smoking	7,934,402	127,730	457,073	54 (90)	0 (0)

*HDL* high-density lipoprotein, *LDL* low-density lipoprotein, *TG* triglycerides, *SBP* systolic blood pressure, *DBP* diastolic blood pressure, *MAP* mean arterial pressure, *PP* pulse pressure, *1df* 1 degree of freedom interaction test, *2df* 2 degrees of freedom joint test, *disc* Discovery stage, *rep* replication stage.

^a^Maximum sample size across all variants analyzed.

^b^Plain text number corresponds to the count of associated loci (region of 1 Mb or more harboring at least one GWAS hit), while the number of independent association signals (associated SNPs remaining after clumping) is provided in parenthesis.

### Identifying single SNP G×E is challenging but accounting for G×E still boost power

Despite the reasonably large sample size available in our studies, there was only one significant interaction signal with the 1df interaction test across the 28 trans-ancestry GWIS when combining discovery and replication (rs1626071 on chr10 near gene *LINC01517*, interaction with current smoking on MAP, *P*_*1df*_ = 3.19 × 10^−8^). For the ancestry-specific meta-analysis, the 1df interaction test identified 8 loci reaching genome-wide significance, all observed in the African ancestry population (Table S2). They involved the smoking exposure only, and are associated with both lipids and blood pressure traits. Four of those loci were also detected at genome-wide significance with the 2df test in the African ancestry population, while the remaining four associations achieve suggestive significance with that test (*P*_*2df*_ between 1.9 × 10^−6^ and 6.3 × 10^−8^).

**Fig. 1:**
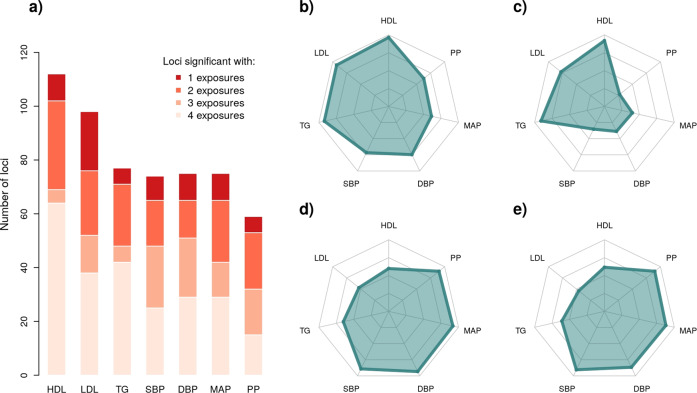
Loci identified by the trans-ancestry 2df joint test across the four exposures. We assessed the relative performance of the trans-ancestry joint 2df test across the four exposures. **a** Overlapping loci for the 2df test across the four exposures. We further decomposed these results by exposure, for current drinking (**b**), drinking habit (**c**), current smoking (**d**), ever smoking (**e**). The corresponding radar plots show the proportion (from 0% to 100%) of the total number of loci identified for that phenotype.

In sharp contrast with the 1df interaction test, the 2df joint test identified a large number of variants in both the trans-ancestry (Table 1) and ancestry-specific (Tables S3 and S4) meta-analyses. Altogether, the 2df trans-ancestry analyses identified a total of 1698 loci-phenotype associations (see “Methods” for the definition of loci), when summing results over all phenotypes and all exposures. Among those, a total of 54% of the loci (*N* = 926) harbored a single independent association signal, while others display multiple independent signals (Fig. S3). Many loci overlapped across the exposures tested. For example, there were 108 and 103 loci identified for HDL when including interaction between current drinking and drinking habits, respectively. However, 92 of those loci were identified in both analyses. Merging all overlapping loci unraveled by different exposure scans, the 2df scans found a total of 112, 98, 77 loci for HDL, LDL, TG, and 74, 75, 75, and 59 loci for SBP, DBP, MAP, and PP respectively. On average, 13% of the loci were identified by a single exposure scan, while 41% were identified by all four exposure association studies for each phenotype (Fig. 1a).

We compared the trans-ancestry 2df results against 599 significant marginal genetic effect association on the two primary blood pressure traits (DBP and SBP) [15–17] and the three lipid traits [20, 27] retrieved from previous studies (Tables S5, S6, Fig. S4, and “Methods”). Among those, 294 were also found genome-wide significant in our studies, and 305 associations did not reach this significance threshold. Conversely, the trans-ancestry 2df screenings identified 119 novel loci-outcome associations. Most of the new association results for lipids were identified when accounting for interaction with drinking exposures, while the majority of new blood pressure associations were identified when accounting for interaction with smoking exposures (Table 2). Part of these observed differences might be explained by heterogeneity in sample size (e.g. *N* was substantially smaller for BP and drinking habits as compared to BP and other exposure). However, sample size for all other phenotype-exposure pairs were fairly very similar (<14%) and unlikely to explain difference in number of replicated signal (e.g. for HDL, *N*_drinking habits_ = 379 K, *N*_current smoking_ = 384 K, and number of replicated signals equals 17 and 11, respectively). We also derived the association signal from the combined stage 1 and 2 SNPs that would have been obtained using a standard marginal genetic effect in the CHARGE data, while adjusting for the effect of the exposure, but not modeling the interaction. The marginal model replicated only at a similar proportion of signal, 48% (*N* = 289) as compared to 49% (*N* = 294) of the 599 previously reported associations. Among the 119 new associations detected by the 2df test, 29% (*N* = 35) did not passed the genome-wide significance level with the marginal model, highlighting the importance of accounting for G×E to detect new associated variants.

**Table 2:** Association signal overlap between the 2df joint test (accounting for interactions) and previous GWAS of marginal genetic effect.

Phenotype	Overall			CHARGE only, per exposure			
	External GWAS only	Both	CHARGE only	Current drinking	Drinking habits	Current smoking	Ever smoking
HDL	70	83	21	18	17	11	11
LDL	60	63	30	25	17	6	6
TG	91	57	15	9	11	6	5
SBP	33	37	35	21	6	27	28
DBP	47	46	26	16	4	22	17
All	301	286	127	89	55	72	67

*HDL* high-density lipoprotein, *LDL* low-density lipoprotein, *TG* triglycerides, *SBP* systolic blood pressure, *DBP* diastolic blood pressure, *1df* 1 degree of freedom interaction test, *2df* 2 degrees of freedom joint test, *disc* discovery stage, *rep* replication stage.

### G×E effects might vary by exposure and ancestry

When stratifying the 2df joint test results by exposure, accounting for interaction with drinking tended to identify more lipids associations, while accounting for interaction with smoking identified more associations for blood pressure phenotypes (Fig. 1b–e). Looking at cross-phenotypes results, GWIS accounting for current drinking and drinking habits captured 81% and 61% of all loci, respectively, and current smoking and ever smoking scans identified 75% and 72% of all loci respectively. Note that the lower number of signals for drinking habits is likely partly explained by the smaller sample size used for that exposure (307 K on average versus 440 K for the other exposures), especially for the BP GWIS that used a different definition for drinking habits (see “Methods”). To understand the differences observed across other exposures, we used the HDL results as a case study. First, we noticed that the chi-squared from the 2df joint test from overlapping loci across the four exposure scans were highly correlated (Fig. S5a). This is expected, as most studies have approximately the same sample size at discovery and replication stages, and contribution of the interaction effect is assumed to be limited. Nevertheless, we noticed a larger mean interaction effect chi-square at those same loci for the drinking exposures ($\overline {\chi ^2}$= 1.57, *P* = 8.7 × 10^−5^ and 1.58, *P* = 1.2 × 10^−4^) as compared to the smoking exposures ($\overline {\chi ^2}$ = 1.07, *P* = 0.32 and 1.09, *P* = 0.28), suggesting a potential contribution of SNP-by-drinking interaction effect (Fig. S5b).

**Fig. 2:**
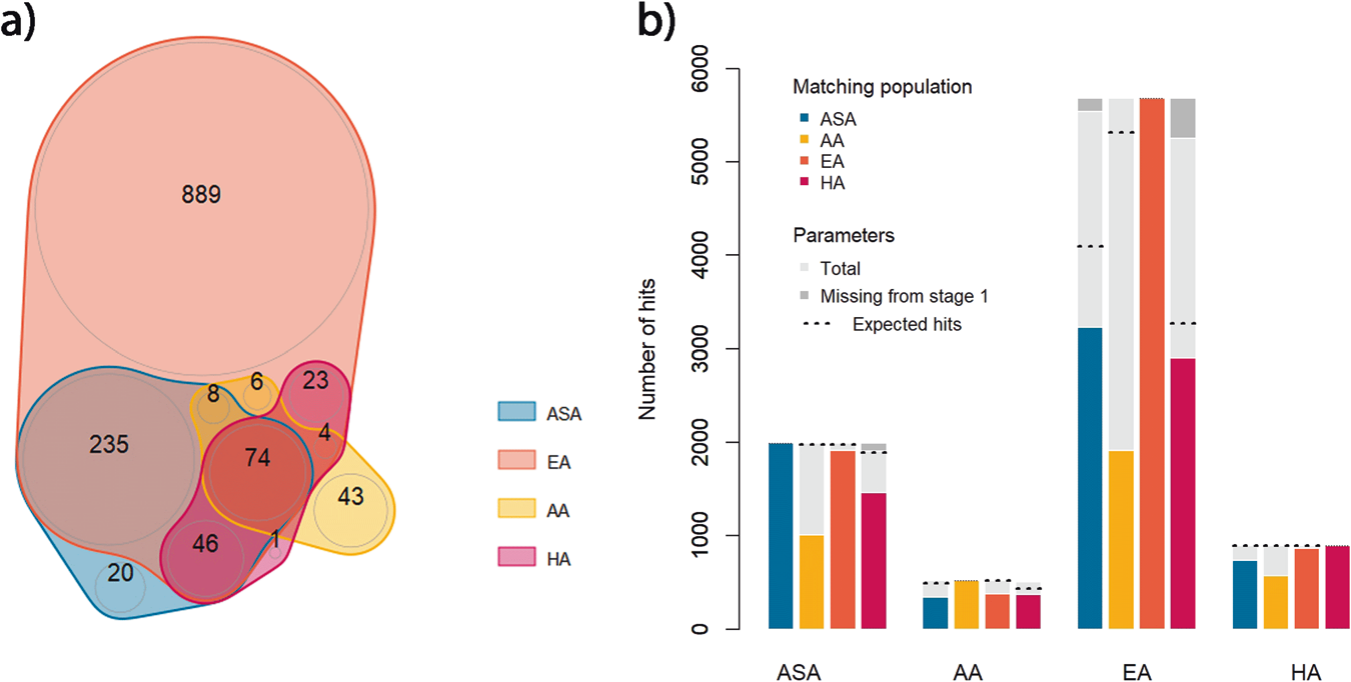
Overlapping associations for the 2df test across ancestries. We derived the overlap in association signal for the joint 2df test of main and interaction effects across the four ancestries: Asian (ASA), African American (AA), European (EA), and Hispanic (HA). **a** A Venn diagram focusing only on loci found at genome-wide significance level after the meta-analysis of stages 1 and 2. In **b** we extracted genome-wide significant SNPs per ancestry (i.e., reference population) after the meta-analysis of stages 1 and 2, and extracted the *p* value for those SNPs in other populations (i.e., the matching populations) from stage 1. The barplot shows for each reference population, the proportion of SNPs in the matching population that achieve a *p* value below 0.05. For each comparison, we also derived the expected number of hits based on the effect size estimate in the reference population and the sample size in the matching population.

Over the two stages, 63% of the individuals (*N* = 380,612) were of European, 27% (*N* = 162,370) of Asian, 6% (*N* = 34,901) of African and 4% (*N* = 22,334) of HAs. For the 2df joint test, the total number of significant associations per ancestry was, as expected, significantly positively correlated to the available sample size (*r*^2^ = 0.42, 95% CI = [0.25, 0.57], *P* = 1.1 × 10^−5^) (Table S3). When merging results from all phenotype-exposure pairs, there were 1,285, 383, 135, and 148 phenotype-variants associations identified after clumping by this approach in EA, ASA, AA, and HA ancestries, respectively. The vast majority of the loci found in the ASA (95%) and HA (99%) ancestries were also identified in the larger EA studies (Fig. 2a). Conversely, 32% (43 out of 135) of the associations identified in AA were exclusively identified in this ancestry and mostly involved variants not present in all ancestries except in AA (in non-AA ancestry populations, 60% of those variants were filtered out at stage 1 because of low frequency). The trans-ancestry analysis identified 1276 (94%) of all ancestry-specific associations, while uncovering an additional 148 associations. All associations missed in the trans-ancestry analyses were found in a single ancestry from ASA (*N* = 6), AA (*N* = 36), EA (*N* = 41), and HA (*N* = 1). To account for sample size differences and assess whether top variants were consistent across ancestries, we extracted the top variants for each ancestry-specific association and checked for nominal significance (*P* < 0.05) in other ancestries screenings from stage 1. Figure 2b shows that the overlap across all phenotypes and per phenotype is modest. These results, along the aforementioned 1df significant signals unique to the AA samples, suggest the presence of ancestry-specific variants and G×E s, and in AA in particular.

### Correlation between marginal genetic and interaction effect are negligible

To understand further the contribution of G×E to significant 2df results, we derived for each phenotype-exposure-ancestry combination (*N* = 12,302, see Table 1 and Table S3) the number of SNPs inducing an enhanced genetic effect in exposed individuals (when main genetic effect and interaction effect have the same direction) and those inducing a reduced genetic effect (when main and interaction have opposite signs). In practice, we used the marginal genetic effect as a proxy for the main effect, as the former parameter has better properties for such an analysis (i.e., the marginal effect is expected to be independent of the interaction effect under the null [12], while the main effect does not [4]). Note that this subtlety has almost no impact on the results as the main and marginal are highly correlated (*ρ* = 0.99) among the significant 2df variants. Overall, the direction of marginal and interaction effects estimated using the 1df test tended to be randomly distributed among those SNPs, although we observed a slight enrichment, with 13 out of 91 trios showing disequilibrium for either concordant or discordant effects (one sided binomial test *P* = 6 × 10^−4^, Fig. S6). Four of them, all in the trans-ancestry analyses, displayed discordant marginal and interaction effect that remained significant after correcting for multiple testing (*P* < 5.5 × 10^−4^): LDL showed larger genetic effects in both current (*P* = 4.3 × 10^−4^) and ever smokers (*P* = 3.7 × 10^−4^), DBP showed larger genetic effects in current drinkers (*P* = 1.6 × 10^−4^), and SBP showed smaller genetic effects among ever smokers (*P* = 9.9 × 10^−5^). Among sets of variants displaying interaction effects discordant with marginal genetic effects, we also searched for those inducing an opposite effect between exposed and unexposed individuals. Although the 2df joint test is supposed to outperform substantially the marginal test in this scenario [4], there were only 66 such associations (0.6% of all associations), suggesting this pattern is quite rare in these data.

**Fig. 3:**
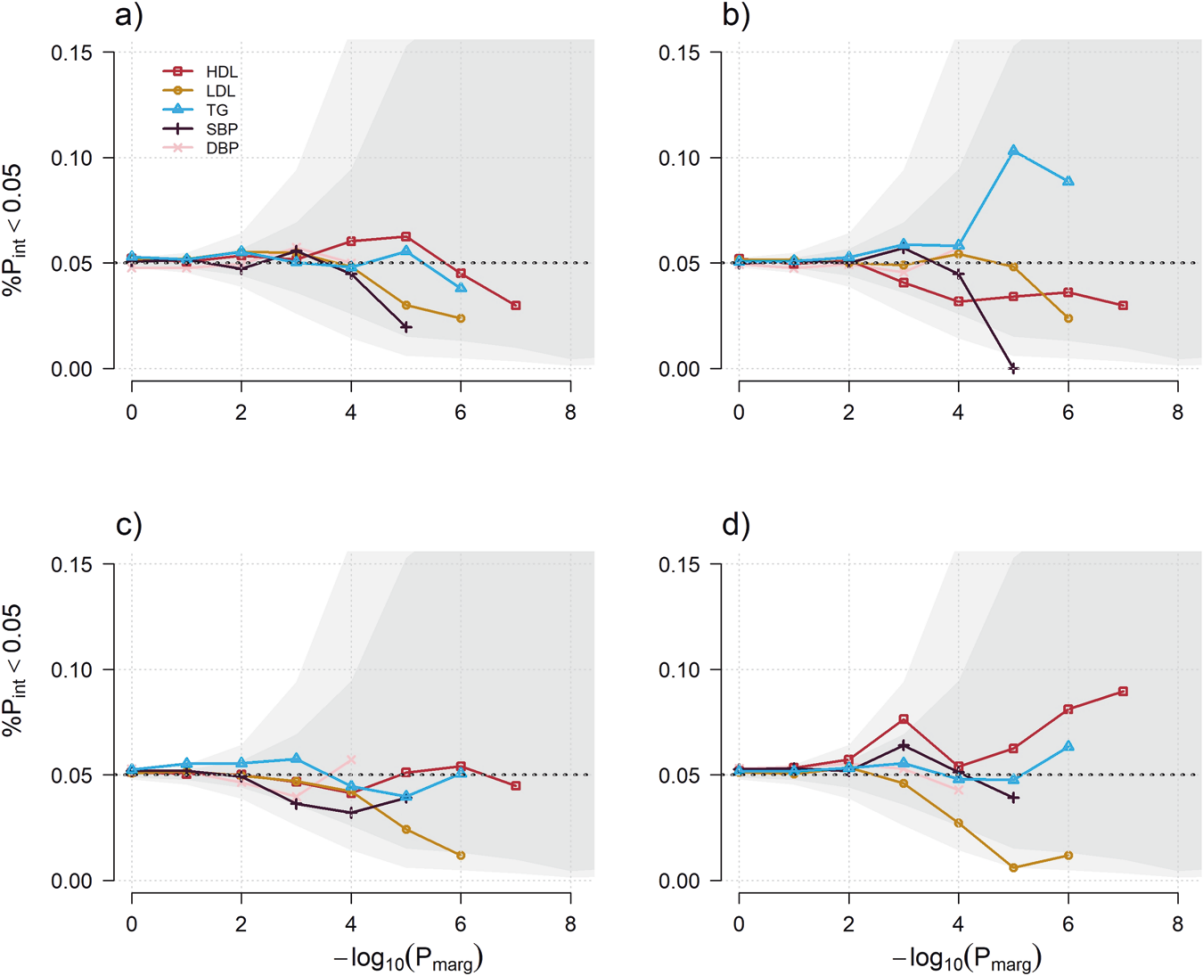
Potential power for 2-step approach. We plotted for each environmental exposure, current drinking (**a**), drinking habits (**b**), current smoking (**c**) and ever smoking (**d**), the proportion of independent SNPs displaying an interaction *p* value (*P*_int_) below 0.05 in CHARGE across bins of variants selected from an independent marginal effect GWAS. Those bins were defined as sets of independent variants with *p* value for marginal genetic effect (*P*_marg_) lower than a given threshold (*x* axis). Each of the five phenotypes are represented by a plain color line. All analyses used stage 1 1df interaction results from European ancestry individuals. Under the null hypothesis of no correlation, the proportion follows a binomial distribution with parameter 0.05 (the black dashed line), independent of the threshold for *P*_marg_. Gray areas indicate the Wilson score confidence interval for an alpha threshold of 0.05 (dark gray) and 1 × 10^−4^ (light gray).

We next assessed potential enrichment for interaction effects across variants previously identified in marginal effect GWAS [15–17, 20, 27] (Tables S5 and S6) and available in the trans-ancestry stage 1 analyses. Among those variants, the smallest single SNP 1df interaction *p* value was observed for rs1260326 (for G×Drinking habits on TG, *P*_*1df*_ = 3.3e−6), a missense variant in *GCKR* previously found associated with alcohol consumption [28, 29]. However, besides this particular signal, the distribution of interaction effects at those variants did not indicate any clear trend (Fig. S7) and the joint test of all single SNP [30] did not find any significant enrichment for interaction effect among these variants (see Supplementary Notes, Table S7). We also explored potential enrichment for interaction at non-significant SNPs. Such enrichment would be of particular interest to increase power of G×E test through 2-step approaches [12, 31, 32] (see for example Fig. S8). The most common 2-step approach consists of filtering out SNPs displaying a marginal genetic *p* value larger than a given α_1_ significance threshold. To assess the potential of this strategy in our data, we quantified the enrichment of nominally significant variants (i.e. *P* < 0.05) for G×E interaction effect while varying α_1_ between 0.1 and 10^−6^ applied to the aforementioned previous marginal GWAS summary statistics. Some phenotype-exposure pairs show a slight increase in the proportion of significant G×E interactions, including in particular TG and drinking habits (11% of the SNPs against the 5% expected for α_1_ = 10^−5^). However, as illutrated in Fig. 3 which display the enrichment along 0.05 and 1 × 10^−4^ confidence interval, no enrichment remains significant after correction for multiple testing in our data. We also considered using marginal genetic effect derived from the stage 1 in CHARGE (Fig. S9). This analysis displayed a modest enrichment for interaction with drinking exposure for lipids and with current smoking for TG, with enrichment for some bins falling outside the stringent confidence interval (i.e., *P* < 1 × 10^−4^).

### Small contribution of G×E at top variants but differences in heritability stratified by exposure

We first used *VarExp*, a tool we recently developed [22], to estimate the variance explained by marginal genetic effects, the joint genetic and G×E interaction effects, and the interaction effects only, at the top genome-wide significant variants in each locus for each phenotype-exposure-ancestry analysis (Table S8). Marginal genetic effects explained between 0.09% and 8.72% of the total phenotypic variance with an average of 3.59%. The fraction of variance explained by the interaction effects only were substantially smaller, varying between 0% and 0.41%, but were statistically significant for many analyses. The largest amount of variance explained was observed for lipids traits, (average of 4.47% for the 2df, as compared to 0.81% for blood pressure phenotypes). Looking at ancestry-specific results, we noted a larger fraction of variance explained in the European ancestry samples than in other ancestries, with greater differences observed in lipids phenotypes and drinking exposures (7.11% of explained variance in individuals from European ancestry versus 5.11% in other ancestries on average). We also noted a slightly higher contribution of G×E in the African ancestry population (0.15%) than in other ancestries (around 0.04%), in agreement with the higher number of significant interactions identified for this ancestry.

**Fig. 4:**
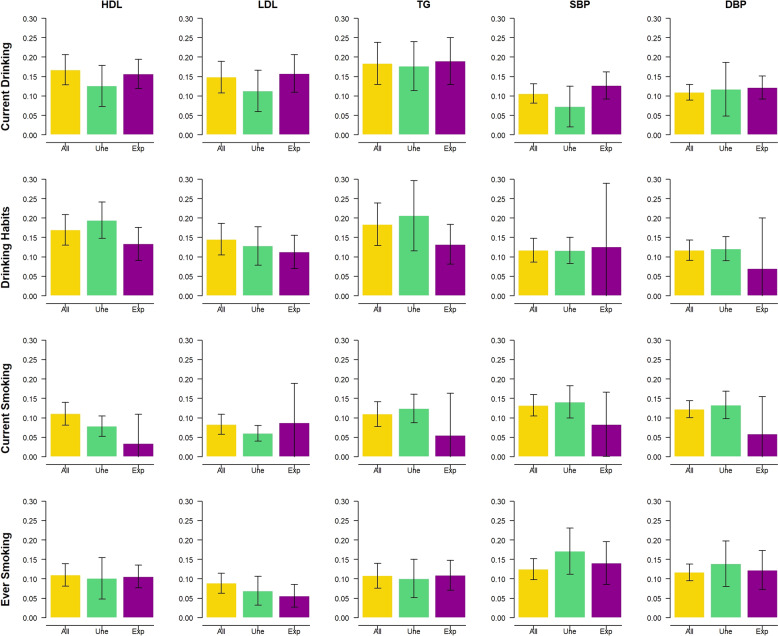
Heritability by exposure group. Heritability of the three lipids and two blood pressure phenotypes (DBP and SBP) derived using the *LDSC* applied to summary statistics from the European ancestry samples meta-analysis. Heritability was derived for all individuals (*All*, yellow bar) and for subset of unexposed (*Une*, teal bar) and exposed (*Exp*, purple bar) individuals. Error bars represent the 95% confidence intervals.

Second, we estimated potential changes in the heritability of the three lipids and two blood pressures (DBP and SBP) traits across all individuals and in strata defined by exposure, using the *LDSC* approach [23] applied to summary statistics from the stage 1 analyses performed in the European ancestry population (Fig. 4, Table S9). Because of potentially biased heritability estimates, we performed a sensitivity analysis, re-deriving the heritability after filtering out SNPs based on their *p* value for heterogeneity in the meta-analysis and selected the most reliable estimate (see Fig. S10 and Supplementary Notes). Based on those estimates, we observed that heritability among exposed individuals was on average smaller than among non-exposed individuals for current smoking ($\overline {h^2}$ = 0.06 and $\overline {h^2}$ = 0.11, respectively) and for drinking habits ($\overline {h^2}$ = 0.12 and $\overline {h^2}$ = 0.15, respectively). Conversely, heritability was on average larger for current drinkers than non-current drinkers ($\overline {h^2}$ = 0.15 and $\overline {h^2}$ = 0.11, respectively). However, only one outcome-phenotype pair showed borderline nominal significance (HDL and drinking habits, with $h^2$ = 0.19, *P* = 7.0 × 10^−17^, and *h*^2^ = 0.13, *P* = 3.1 × 10^−10^ for unexposed and exposed, respectively, *P* = 0.052), and this difference did not remain statistically significant after correction for multiple testing.

### Differential pathways across exposures

To explore further differences in genetic effect between exposure strata, we partitioned the genetic heritability estimated in individuals from European ancestry across different functional annotations [25, 26]. We first considered baseline annotations provided with the *LDSC* package and the *GenoSkyline* [33] annotation set, a cell-type-specific annotation database derived mainly from the Roadmap Epigenomics [34] (Figs. S11–15). Because of the relatively modest sample size in some strata (*N* = 12,578 in the smallest strata, see Table S1), we focused on the distribution of the estimated enrichment coefficient between exposed and unexposed. The majority of phenotype-exposure pairs exhibited a similar enrichment pattern (Fig. S16). For example, the enrichment estimates were significantly correlated for drinking habits exposure and lipids (correlations equal 0.76 (*P* = 9.5 × 10^−24^), 0.60 (*P* = 5.8 × 10^−13^) and 0.40 (*P* = 7.7 × 10^−6^) for HDL, LDL and TG, respectively), suggesting that potential G×E interactions for those phenotypes do not involve new pathways. Conversely, LDL shows substantial variability in enrichment for the three other exposures (correlations equal 0.10 (*P* = 0.25), 0.22 (*P* = 0.01), and 0.17 (*P* = 0.07), for current drinking, current smoking, and ever smoking, respectively), suggesting those exposures might activate new genetic pathways while reducing the effect of genetic variants involved in unexposed individuals. We also noted substantial variability for the phenotypes-exposure pairs showing the largest differences in heritability (lipids and current smoking, and BP and drinking habits, see Fig. 4). However, part of that variability might be due to the reduced sample size in one of the two strata, thus making interpretation challenging.

**Fig. 5:**
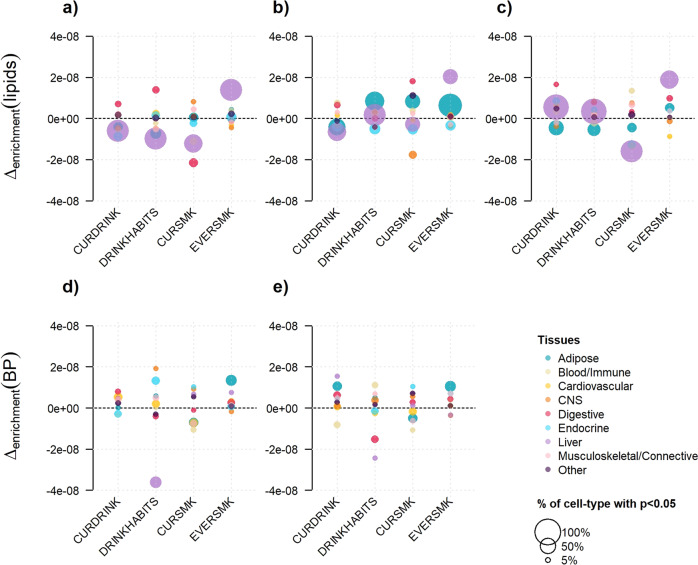
Stratification of heritability by tissue. Cell-type partitioned heritability for each exposure was performed and further merged into nine primary tissue categories. The top panels show the results for lipids: LDL (**a**), HDL (**b**), and TG (**c**), and the bottom panels show the results for blood pressure: DBP (**d**), and SBP (**e**). For each phenotype-exposure pair we derived the difference in enrichment defined as the median enrichment in unexposed minus the median enrichment in exposed individuals (Δ_enrichment_) per tissue. To highlight the significance of enrichment within each cell type, we scaled the size of each data point by the proportion of cell types that are nominally significant (i.e., *P* < 0.05) after merging exposed and unexposed results.

We next investigated whether exposures tended to display systematic enrichment in specific tissues [26]. For each phenotype, heritability was stratified based on annotation from 205 cell types linked to 9 tissues (adipose, blood/immune, cardiovascular, central nervous system, digestive, endocrine, liver, musculoskeletal/connective, and other), in unexposed and exposed individuals separately (Fig. 5, Figs. S17–S21). Because of unbalanced sample size between strata, we focused on the relative differences in median enrichment between exposed and unexposed by tissue, and reported the proportion of cell types nominally significant for enrichment in each tissue. Overall, liver and adipose were the most enriched and most significant tissues for lipids traits, while showing variability between exposed and unexposed individuals. LDL also showed some significance and variability for cell types mapped to digestive tissue for the drinking exposures and current smoking (Fig. 5a). There was less significant enrichment and a less marked difference for BP traits, although we noticed a substantially larger enrichment in liver tissue among heavy drinkers versus low-drinkers for DBP (Fig. 5d).

### Genetic heterogeneity, power, and risk of bias for the test of interaction in trans-ancestry analysis

Throughout this work, we used G×E interaction effect estimates and *p* values derived using the standard 1df inverse-variance meta-analysis scheme as described in Willer et al. [35], and applied to the 1df interaction effect from each contributing cohort similarly to the original GWIS papers. On the other hand, we used the main genetic effect estimates and *p* values derived from the 2df framework as described in Manning et al. [36]. Although, the 2df framework provides a joint estimation of the main and interaction effect coefficients along with standard errors, we did not use the interaction effect parameters from that model as we identified potential biases in those estimates through several simulation studies (see Supplementary notes for more details and Table S10). Heterogeneity for both the main genetic effect and the proportion of exposed individuals between the two cohorts (e.g., higher genetic effect in one cohort combined to a higher level of exposure) can bias the interaction effect estimates in the 2df framework and result in a severe type I error rate inflation, inducing false-positive associations (Fig. S22). However, main genetic effects estimates are not biased in the 2df framework in the case of a binary exposure (Fig. S23) but can be noisy in the case of a continuous exposure (Fig. S24). These simulation studies highlighted the special care required to interpret results from the 2df framework.

## Discussion

In this study, we assembled and synthesized the results from 28 G×E interaction GWIS on lipid and blood pressure phenotypes performed across four ancestries. Overall, we found the trans-ancestry 2df test to be efficient for SNP discovery, with the vast majority of associations identified in ancestry-specific analyses being confirmed in the trans-ancestry analysis, while allowing for a 10% increase in detection. However, our data also pointed toward ancestry-specific patterns for interaction effects, especially for African ancestry populations. Differences were also observed when comparing results across exposures. We noted a greater increase in detection for lipid-associated variants when accounting for interaction with drinking, and a greater increase in detection for blood pressure-associated variants when accounting for interaction with smoking. When leveraging marginal genetic effect reported from previous studies to select potential candidates for interaction effects, we did not observe any significant enrichment for interaction effects whatever the significance level used. This is in agreement with our in-depth comparison of main genetic and interaction effects using the consortium data, which found only modest correlation between the interaction and main effects coefficients. Finally, our assessment of variance explained by interaction effects suggests that, even if small, accounting for interaction can help push signals above the stringent genome-wide significance threshold. Furthermore, the stratification of heritability by functional annotations highlighted that exposures can induce divergent mechanisms of phenotype production with modification in the associated genetic pathway and cell type involved.

Our estimation of the phenotypic variance explained by marginal genetic effect and interaction shows, in agreement with previous studies, that the contribution of G×E terms on top of marginal genetic effect is relatively modest. It confirms the likely limited impact of discovering G×E for prediction purposes in the general population [37]. The variability between non-smokers and drinkers observed in the exposure-specific heritability is intriguing, but might potentially be explained by other factors which cannot be sorted out using these data. Further work is needed not only to understand this heterogeneity but also to assess potential gain in predictive power of polygenic risk score derived by exposure strata [38]. Importantly, a modest contribution of G×E to phenotypic variance does not rule out the potentially important role of G×E in the etiology of these traits. And, for example, our stratified heritability analyses suggest a potential change in the genetic architecture of LDL conditional on smoking and BP traits conditional on current drinking.

The statistical power of the GWIS varied substantially across analyses. Taking the average sample size across all phenotype-exposure pairs analyzed per ancestry, there was 80% power at an alpha threshold of 5 × 10^−8^ to detect interaction effect explaining 0.0096% (trans-ancestry, $\bar N$ = 440 K), 0.016% (EA, $\bar N$ = 271 K), 0.15% (AA, $\bar N$ = 27 K), 0.034% (ASA, $\bar N$ = 123 K), and 0.22% (HISP, $\bar N$ = 19 K) of the outcome variance. The observed enrichment for interaction effects in AA as compared to other ancestries is therefore quite striking, and further investigation in larger data is required. It would also be of interest to explore whether interactions play a role in the well documented differences in prevalence of both blood pressure outcomes (e.g. hypertension [39]) and lipids (e.g. low HDL [40]) in individuals from African-American ancestry. The sample size was also critical when deriving heritability. Here, we only considered the European ancestry data as sample size for other cohorts was too small to derive meaningful estimates. Nevertheless, statistical power remains limited in EA for *LDSC* stratified analyses based on functional annotation, and future larger studies are also required to validate the observed enrichments.

We fully appreciate that the results from the several experiments we conducted are challenging to aggregate into a single uniform framework. Our analysis rather suggests first that even though related traits share some features; they can also display substantial heterogeneity at other levels. For example, all lipids harbor more signals when accounting for drinking exposures, but at the same time display very different patterns when investigating functional enrichment. It also suggests that the links between heritability, genetic mechanisms involved, and the resulting distribution of G×E effect across SNPs are not straightforward. Finally, our careful assessment of each step of the analyses highlights that complexity also shows up at the methodological level, with a potential for introducing bias at several stages, and so the extra care needed for interpretation. Despite those limitations, we argue that systematic and careful evaluation of G×E across multiple phenotype-exposure-ancestry combinations, as done in this study, still provides critical insight of the interplay between genetic and environmental factors, offering long-term opportunities for numerous additional follow-up analyses down to the biological mechanisms underlying the phenotypes and their interaction with the environment.

## Supplementary information

Supplementary Tables

Supplementary Notes and Figures

## Data Availability

All of the data used in this work are publicly available. Both the original GWAS summary results and the re-processed statistics generated as part of this study are available via dbGaP (accession number phs000930).
