# Supplementary material for: Gene-lifestyle interactions in the genomics of human complex traits
Source: Eur J Hum Genet. 2022 Mar 22;30(6):730–9. doi: 10.1038/s41431-022-01045-6 (PMC9178041; doi:10.1038/s41431-022-01045-6)
Supplement: Supplementary file 2 — Supplementary Notes and Figures [file 41431_2022_1045_MOESM2_ESM.docx]

**Gene-lifestyle interactions in the genomics of human complex traits.**

**Supplementary data**

Table of Contents

[Supplementary Note 2](#_Toc83030505)

[Phenotypes and exposures 2](#_Toc83030506)

[Data processing 2](#_Toc83030507)

[Test interaction 1DF and 2DF and meta-analysis 3](#_Toc83030508)

[Joint test of interaction across multiple SNPs. 4](#_Toc83030509)

[Variance explained at top SNPs. 4](#_Toc83030510)

[Heritability 5](#_Toc83030511)

[Stratified heritability 5](#_Toc83030512)

[Simulation study 6](#_Toc83030513)

[Integration of external data 6](#_Toc83030514)

[Supplementary Figures 8](#_Toc83030515)

[Figure S1 QQplots 8](#_Toc83030516)

[Figure S2. Proportion of exposed individuals 9](#_Toc83030517)

[Figure S3. Distribution of independent signals per locus 10](#_Toc83030518)

[Figure S4. Comparison of the 2df joint test in CHARGE against external marginal effect GWAS 11](#_Toc83030519)

[Figure S5. Comparison of HDL results 12](#_Toc83030520)

[Figure S6. Marginal and interaction effects among SNPs significant with the 2df test 13](#_Toc83030521)

[Figure S7. Interaction effect at previously reported loci 14](#_Toc83030522)

[Figure S8. Example of power for 2 df interaction test 15](#_Toc83030523)

[Figure S9. Potential power for 2-step approach 16](#_Toc83030524)

[Figure S10. Variability in heritability estimation 17](#_Toc83030525)

[Figure S11. Annotation enrichment for DBP 18](#_Toc83030526)

[Figure S12. Annotation enrichment for SBP 19](#_Toc83030527)

[Figure S13. Annotation enrichment for HDL 20](#_Toc83030528)

[Figure S14. Annotation enrichment for LDL 21](#_Toc83030529)

[Figure S15. Annotation enrichment for TG 22](#_Toc83030530)

[Figure S16. Cell-type enrichment for DBP 23](#_Toc83030531)

[Figure S17. Cell-type enrichment for SBP 24](#_Toc83030532)

[Figure S18. Cell-type enrichment for HDL 25](#_Toc83030533)

[Figure S19. Cell-type enrichment for LDL 26](#_Toc83030534)

[Figure S20. Cell-type enrichment for TG 27](#_Toc83030535)

[Figure S21. Example of power for 2 df interaction test 28](#_Toc83030536)

[Figure S22. Robustness of the 2df framework for binary exposures 29](#_Toc83030537)

[Figure S23. Robustness of the 2df framework 30](#_Toc83030538)

[Figure S24. The joint test can capture genetic heterogeneity 31](#_Toc83030539)

[References 32](#_Toc83030540)

# Supplementary Note

## Phenotypes and exposures

We considered four blood pressure phenotypes (DBP, SBP, PP, MAP), and three lipids levels (TG, HDL, LDL). DBP and SBP were derived as the average over multiple measurements performed at resting or sitting positions. PP and MAP were derived as the difference between SBP and DBP, and the sum of two-thirds of DBP and one-third of SBP, respectively. In all cohorts, HDL and TG were directly assayed, while LDL was either directly assayed or estimated using the Friedewald equation(1): LDL = TC − HDL − (TG/5). Both HDL and TG were natural log transformed, while LDL was not transformed. Additional details of the phenotype transformation have been published here(2).

Two binary smoking exposures, *current smoking* and *ever smoking*, were considered and measured similarly across all smoking GWIS. The *current smoking* variable was coded as 1 if the subject smoked regularly in past year and as 0 otherwise. *Ever smoking* status was coded as 1 if the subject smoked at least 100 cigarettes during his/her lifetime and 0 otherwise. For alcohol consumption, two binary variables were considered, referred further as *current drinking* and *drinking habit*. For both blood pressure and lipid traits, the former exposure was defined similarly for all studies, corresponding to any recurrent drinking behavior. Conversely, the *drinking habits* exposure was defined differently across publications. For lipids phenotypes, the variable was coded as 1 for the subset of current drinkers having at least two drinks per week and 0 for everyone else (*i.e.,* the no drinkers and those drinking less than two drinks per week)(3). The blood pressure GWIS used instead a “low *versus* heavy drinking”, where the variable was coded 1 for individuals having at least 8 glasses per week, and 0 for individuals with less than 8 glasses per week, while all non-drinkers were removed(4). Because of this heterogeneity, we limited the cross-phenotypes comparison per exposure, and for drinking habits in particular.

Generally, the use of categories for the exposures instead of continuous exposure data was necessary for harmonizing data across the many cohorts included in the original meta-analyses. Additional details on the assessment of the exposure and phenotypes are provided in the corresponding publications(3-7).

## Data processing

All studies conducted a two-stage approach. In stage 1 (referred to as *Discovery*), a standard GWIS was performed using up to 18 million genetic variants. In stage 2 (referred to as *Replication*), only a subset of variants with a *p*-value for either 1df or the 2df test below a certain threshold (P<10^-6^ or P<10^-5^) at stage 1 were further considered. More details can be found in the corresponding publications(3-7). For each outcome-exposure, we had access to complete meta-analysis summary statistics of both the discovery and the replication stages for four different ancestries (European, African, Asian and Hispanic) after quality control filtering. To ensure a fair comparison, we re-processed all results for each outcome-exposure-ancestry combination using the same pipeline. In the discovery stage, we excluded SNPs with a MAF below 1% and with significant (*P* < 10^-6^) heterogeneous effects across cohorts. SNPs present in only one ancestry were excluded from trans-ancestries analyses. Trans-ancestry summary statistics in the replication stage were filtered similarly to the discovery stage. Finally, we computed meta-analyses results for the combined analyses (discovery stage + replication stage) in each individual ancestry and trans-ancestry. When describing genome-wide significant results for each ancestry and each phenotype-exposure combination, only SNPs included in both stages were considered. All meta-analyses were computed using the inverse-variance scheme for the 1df interaction test and the 2df framework for the 2df joint test implemented in the METAL software(8).

When deriving shared associated loci across studies (*e.g.,* comparing results from a given phenotype across exposures or ancestries), we merged the overlapping ones, resulting in total counts of associated loci sometimes slightly lower than the expected total. For example, two loci close to each other in one analysis might overlap with a single locus from another analysis. In such case, the two former loci were merged into a single (larger) locus.

## Test interaction 1DF and 2DF and meta-analysis

For a SNP $G$, an exposure $E$, and a set of covariates $\mathbf{C}$, the GWIS test conducted at each stage in all studies can be expressed as:

$$Y \sim\beta_{g} G + \beta_{e} E + \beta_{ge} G\times E+ \boldsymbol{\beta}_{\boldsymbol{C}} \boldsymbol{C}$$

The standard 1df test of interaction evaluate $\hat{\beta}_{ge}$, while the 2df test evaluate jointly $\hat{\beta}_{g}$ and $\hat{\beta}_{ge}$. Independent of the specific exposure considered for interaction, all models included adjustment for all four exposures, along other relevant risk factors.

We then used METAL(8, 9) to meta-analyze results from all individual cohorts. In its implementation, METAL allows to perform meta-analyses for the 2df joint test described above. Briefly, let $\hat{\beta}_{g,i}$ *and* $\hat{\beta}_{ge,i}$ denote the main genetic effect and the interaction effect estimated in the cohort $i$ ($i=1\cdots N$), $\hat{\Sigma_{i}}=cov\left( \hat{\beta}_{g,i},\hat{\beta}_{ge,i} \right)$ the variance-covariance matrix of the main genetic and interaction effects in cohort $i$. Then, the meta-analysed effects $\hat{\beta}=(\hat{\beta}_{g}, \hat{\beta}_{ge})$can be computed by:

$\hat{\beta}=\left( W^{T}\Sigma^{-1}W \right)^{-1}W^{T}\Sigma^{-1}b$ and $cov\left( \hat{\beta} \right)=$ $\left( W^{T}\Sigma^{-1}W \right)^{-1}$

with $b=\left( \hat{\beta}_{g,1},\hat{\beta}_{ge,1},\cdots,\hat{\beta}_{g,N},\hat{\beta}_{ge,N} \right)^{T}$, $\Sigma=\left( \begin{matrix} \hat{\Sigma_{i}} & 0 & \ldots& 0 \\ 0 & \ddots& 0 & 0 \\ \vdots& 0 & \ddots& \vdots\\ 0 & 0 & \ldots& \hat{\Sigma_{N}} \end{matrix} \right)$ and $W=\left( \begin{matrix} 1 & 0 \\ 0 & 1 \\ \vdots& \vdots\\ 1 & 0 \\ 0 & 1 \end{matrix} \right)$.

The Wald statistic used to evaluate $\hat{\beta}=\left( \hat{\beta}_{g}, \hat{\beta}_{ge} \right)=0$ is $\beta^{T}{cov\left( \hat{\beta} \right)}^{-1}\beta$ and follows a Chi-square distribution with two degrees of freedom.

To compute 1df interaction effect combined across all individual cohorts, we performed a standard inverse-variance weighted meta-analysis of the interaction effects $\hat{\beta}_{ge, i}$,. obtained in each individual cohorts:

$\hat{\beta}_{ge}=\sum_{i=1}^{N} \frac{w_{i}\hat{\beta}_{ge,i}}{\sum_{i=1}^{N} w_{i}}$ and $\hat{\sigma}_{\hat{\beta}_{ge}}=\sqrt{\frac{1}{\sum_{i=1}^{N} w_{i}}}$

where $w_{i}=\frac{1}{var\left( \hat{\beta}_{ge,i} \right)}$.

The Wald statistic defined as $\left( \frac{\hat{\beta}_{ge}}{\hat{\sigma}_{\hat{\beta}_{ge}}} \right)^{2}$ follows a Chi-square distribution with one degree of freedom and is used to test $\hat{\beta}_{ge}=0$.

When comparing interaction models against marginal genetic models in CHARGE, we consider the marginal model adjusted for the main effect of all four exposures:

$$Y \sim\beta_{g} G + \boldsymbol{\beta}_{\boldsymbol{e}}\boldsymbol{E} + \boldsymbol{\beta}_{\boldsymbol{C}} \boldsymbol{C}$$

where $\boldsymbol{\beta}_{\boldsymbol{e}}$ and $\boldsymbol{E}$ are the vector of effect for the four exposures and the four columns matrix of exposure values.

Similarly, genetics effects in unexposed and exposed individuals were estimated using a marginal model adjusted for the exposure and other relevant covariates.

When the marginal effect was unavailable, we derived the corresponding summary statistics from the interaction model using the J2S tool we recently developed(10). Briefly, marginal genetic effects $\hat{\beta_{marg}}$ in all individuals, $\hat{\beta_{unexp}}$in unexposed and $\hat{\beta_{exp}}$ in exposed individuals separately can be derived using the summary statistics from the joint model with interaction as follows:

$$\hat{\beta_{marg}}= \hat{\beta_{g}}+\hat{\beta_{ge}}\mu_{E}; \sigma_{\hat{\beta_{marg}}}=\sqrt{\sigma_{\hat{\beta_{g}}}^{2}+\mu_{E}^{2}\sigma_{\hat{\beta_{ge}}}^{2}+2\mu_{E}cov\left( \sigma_{\hat{\beta_{g}}},\sigma_{\hat{\delta}} \right)}$$

$$\hat{\beta_{unexp}}=\hat{\beta_{g}}, \sigma_{\hat{\beta_{unexp}}}=\sigma_{\hat{\beta_{g}}}$$

$\hat{\beta_{exp}}=\hat{\beta_{g}}+\hat{\beta_{ge}}, \sigma_{\hat{\beta_{exp}}}=\sqrt{\sigma_{\hat{\beta_{g}}}^{2}+\sigma_{\hat{\beta_{ge}}}^{2}+2cov\left( \sigma_{\hat{\beta}},\sigma_{\hat{\beta_{ge}}} \right)}$

Where $\mu_{E}$ denotes the proportion of exposed individuals.

## Joint test of interaction across multiple SNPs.

Consider a vector of $L$ single SNP interaction coefficient $\boldsymbol{S}=\left\lceil\hat{\gamma}_{1},\ldots\hat{\gamma}_{L} \right\rceil$ and their corresponding variance-covariance matrix $\mathbf{Q}$, which off-diagonal term equal 0, and diagonal terms are the variance of each estimate $\boldsymbol{\Gamma}=\left[ \sigma_{\hat{\gamma}_{1}}^{2},\ldots\sigma_{\hat{\gamma}_{L}}^{2} \right]$. In the standard omnibus test, all interaction effects, are tested jointly, by forming the statistics $\boldsymbol{S}^{\boldsymbol{T}}\mathbf{Q}^{-1}\boldsymbol{S}$, which follows a chi-square distribution with L degree of freedom. For GRS-based interaction tests, we assume the GRS are built as the (weighted or unweighted) sum of risk alleles of the $L$ candidate SNPs. Explicitly, $uGRS =\sum_{i=1\ldots L} G_{i}$, and $wGRS=\sum_{i=1\ldots L} w_{i}\times G_{i}$, where $w_{i}$ is the marginal genetic risk estimate of SNP $i$. We aim at testing the significance of the $uGRS\times E$ and $wGRS\times E$. As previously demonstrated(11), the corresponding statistical tests can be respectively approximated using interaction summary statistics as ${\left( \sum_{i=1\ldots L} \left( {\hat{\gamma}_{i}}/{\sigma_{\hat{\gamma}_{i}}^{2}} \right) \right)^{2}}/{\sum_{i=1\ldots L} \left( 1/{\sigma_{\hat{\gamma}_{i}}^{2}} \right)}$, and ${\left( \sum_{i=1\ldots L} \left( {w_{i}\hat{\gamma}_{i}}/{\sigma_{\hat{\gamma}_{i}}^{2}} \right) \right)^{2}}/{\sum_{i=1\ldots L} \left( {w_{i}^{2}}/{\sigma_{\hat{\gamma}_{i}}^{2}} \right)}$, and both follow a 1 degree of freedom chi-square under the null.

## Variance explained at top SNPs.

We estimated the fraction of phenotypic variance explained by the main effects, the interaction effects and those effects jointly $f_{G}$, $f_{I}$, $f_{J}$ respectively using the R package *VarExp*^(12)^. Considering a joint regression model including interaction terms fitted in a sample of $N$ individuals, the fraction of phenotypic variance explained by the genetic main effects of a set of SNPs, their interaction effect and jointly can be estimated using only summary statistics from the joint model by $f_{G}=\frac{N\left( {\alpha_{G}^{'}}^{T}\Sigma^{-1}\alpha_{G}^{'} \right)-q}{\left( N-q \right)var\left( Y \right)}$, $f_{I}=\frac{N\left( {\alpha_{INT}^{'}}^{T}\Sigma^{-1}\alpha_{INT}^{'} \right)-q}{\left( N-q \right)var\left( Y \right)}$ and $f_{J}=\frac{N\left( {\alpha_{G}^{'}}^{T}\Sigma^{-1}\alpha_{G}^{'} + {\alpha_{INT}^{'}}^{T}\Sigma^{-1}\alpha_{INT}^{'} \right)-q}{\left( N-q \right)var\left( Y \right)}$ respectively; where $\alpha_{G}^{'}$ and $\alpha_{INT}^{'}$ denote the standardized main genetic and interaction effects and $q$ is the rank of the SNP correlation matrix $\Sigma$.

This analysis was conducted in each ancestry for each phenotype-exposure combination separately, using only genome-wide significant SNPs in the combined meta-analyses for either the 2df or the 1df test for the given trio (exposure-phenotype-ancestry). For simplicity, we clustered SNPs into loci of 1Mb (500kb from the top SNP upstream and downstream) and computed the variance explained using only the lead SNPs (with the lowest p-value) for all loci. Also, because of potentially biased estimations of the interaction effect sizes using the 2df framework but not for the main genetic effect size (*see* last result section and **Figure S22**), we used the genetic main effect size estimates from the joint framework and the interaction effect sizes computed using the standard 1df meta-analyses for the interaction test

## Heritability

We computed the genetic heritability from the summary statistics of the different screenings using the LD Score. Briefly, regressing the Wald test statistics ($\chi^{2})$against the LDscores ($l)$ which quantify the amount of linkage disequilibrium of a SNP with other variants provides an estimate of the genetic heritability $h^{2}$ as $\mathbb{E}\left[ \chi_{j}^{2}|l_{j} \right]={Nh^{2}l_{j}}/M+Na+1$, where $M$ is the number of SNPs, $N$ is the sample size and $a$ quantifies the contribution of confounding factors.

To avoid genetic heterogeneity issues, we focused on European ancestry samples only. We computed the genetic heritability based on the marginal genetic effect in the whole sample (*i.e.* unexposed and exposed combined) and based on marginal genetic effect in exposure-specific strata (*i.e.* unexposed and exposed individuals separately) for each trait and exposure combination using the *LDSC* approach(13). We used the pre-computed *LDSC* relative to European ancestry samples provided with the software. When unavailable from the original studies, we derived the summary statistics of the genetic marginal effect in the whole sample and in unexposed and exposed individuals from the interaction model using a tool we recently developed(10)

A metric indicating the validity of the computation is the ratio between the intercept of the regression and the mean Chi-square $\chi^{2}$. This ratio measures the proportion of the inflation in the mean $\chi^{2}$ that the intercept from the regression model ascribes to causes other than polygenic heritability. This ratio is expected to be close to 0 while values substantially deviating from 0 indicate likely poor estimations of the genetic heritability due to a potential mismatch between the sample and the reference population or model specifications. In our studies, we observed some ratios largely deviating from 0. We therefore conducted a sensitivity analysis by estimating the genetic heritability after filtering SNPs in our study based on their p-values for the test of heterogeneity across cohorts or population. In practice, we kept SNPs with a p-value higher than a given threshold (P>0, >0.3, >0.5, >0.8, >0.9) and computed the genetic using this set of SNPs. Finally, we retained the estimation from the set of SNPs leading to the smallest ratio and a number of included SNPs greater than 200,000.

Significance of the observed difference in heritability was derived using a two-sided, two samples z-test which the statistics, defined as $\left( h_{e}^{2}-h_{u}^{2} \right)/{\sqrt{\sigma_{e}^{2}+\sigma_{u}^{2}}}$ where $h_{e}^{2}$and $\sigma_{e}^{2}$ (resp. $h_{u}^{2}$and $\sigma_{u}^{2}$) are the heritability and standard error in exposed (resp. unexposed) individuals, follows a scaled normal distribution. Differences with a p-value below the Bonferroni corrected threshold (0.05 / 20 = 2.5 x 10^-3^) were considered as significant.

## Stratified heritability

For each exposure stratum, genetic heritability, derived from all available SNPs, was partitioned by both cell type-specific and general annotations(14). We used two distinct sets of annotations: *baseline* and *Genoskyline+.* The baseline annotations encompass 53 tissue-agnostic, general functional annotations. Annotations in this set are binary and are not mutually exclusive; each variant is labeled with an indicator variable for each annotation and may belong to multiple annotation classes. GenoSkyline+ is a recently proposed annotation set integrating a rich collection of epigenomic data from the Roadmap Epigenomics Project(15). The suite of annotations are tissue-specific, genome-wide predictions of functionality, generated through integrative analysis of epigenetic and gene expression. Unsupervised learning methods were used to generate posterior probabilities of functionality at every genomic coordinate within the human genome for 127 tissue-specific annotations. The authors provide pregenerated data for use with the LDscore software. For each Hapmap3 variant, each annotation is dichotomized by assigning a positive indicator if the posterior probability is greater than or equal to 0.5, and a negative indicator otherwise.

Enrichment and annotation-specific heritability were compared across exposure strata for each trait. When assessing the significance of the enrichment, we used a Bonferroni corrected significance threshold of P < 0.000277. We further quantified enrichment for tissue-specific heritability following Finucane et al.(16), where results from cell-specific annotations based on gene expression data were gathered into tissue-specific classes. Except when specified otherwise, enrichment analyses compared median enrichment between exposure strata. We avoided comparison of significance level which would be biased by differences in sample size.

## Simulation study

We compared the performances of meta-analysis strategies for estimating and testing the main and interaction effects across multiple cohorts from the interaction model. The first strategy (1df framework) uses the effect estimate and standard error of the parameter of interest (either the main or interaction term) from each individual cohort and then performs a standard 1 degree of freedom inverse-variance weighted meta-analysis. The second strategy (2 df framework) performs first a meta-analysis of both parameters jointly, using not only single cohort effect estimates and standard errors, but also their covariance(8, 17). It then uses the effect estimates from the previous step to perform a standard *Wald* test of each parameter separately.

We conducted two series of simulations, all including two cohorts for simplicity. The first one focused on understanding differences we observed for the interaction effect between the two frameworks on real data. We generated 20,000 genotypes per cohort, a binary exposure and a phenotype as a linear combination of a main genetic effect, an interaction effect, or both. These simulations aimed at assessing the impact of heterogeneity across cohorts, when varying the MAF of the SNPs, the proportion of exposed individuals in the two cohorts, and the effect sizes of the main genetic effect and of the interaction effect. For each replicate and each scenario, we performed linear regressions testing for interaction between the SNP and the exposure in each cohort separately and applied the two aforementioned frameworks to meta-analyze the interaction parameter. Among the many possible scenarios, we focus on a subset of 24 illustrating the key factors inducing bias. In most cases, *p*-values for interaction computed using the two frameworks were asymptotically similar. However, we found that heterogeneity for both the main genetic effect and the proportion of exposed individuals between the two cohorts (*e.g.* higher genetic effect in one cohort combined to a higher level of exposure) can bias the interaction effect estimates in the 2df framework (scenarios 18, 20, 22 and 24 in **Figure S22**). In the complete absence of interaction effect this bias can result in a severe type I error rate inflation. Even though heterogeneity in the main genetic effect might be explained by a form of GxE interaction, it can also likely be due to differences in linkage disequilibrium between causal and typed variants –a realistic situation when meta-analyzing studies from diverse ancestries– and thus inducing a false interaction signal.

The second simulation broadened the scope of the assessment, and compared estimated coefficients and corresponding chi-squared test for both the interaction and main genetic effect terms for the two frameworks. Here, we simulated a series of 1,000 replicates using either a binary outcome or a continuous outcome and a single SNP, while varying all parameters (distribution of the exposure, MAF, presence and size of the genetic and interaction effects, sample size of each cohort) at random and independently between the two cohorts. We also used a similar framework to compare the power and robustness of four alternative model: i) the 1df marginal genetic effect model only testing the effect of the genetic variants (Y~G), ii) the same model but adjusting for the effect of the exposure (Y~G+E), iii) a joint 2df model accounting for interaction between G and E, as used in our studies (Y~G+E+GxE), and iv) an alternative joint 2df accounting for interaction between G and the cohort status (Y~G+C+GxC). The use of the 2df framework accounting for GxE interaction can substantially boost power to detect variants despite the absence of such interaction (**Figure S25)**. This is explained by the exposure acting as a proxy for the cohorts, which itself involved a statistical interaction because of the genetic heterogeneity. Indeed, the 2df joint test accounting for GxCohort interaction shows the highest power in this scenario. Altogether this highlighted the special care required to interpret results from the 2df framework.

## Integration of external data

We used several external data in our analyses. The specificity of our study (multi-ancestry, gene-environment interaction, two-stage analysis) made the integration of those external data challenging. For example, the partial replication rate with independent marginal genetic effect GWAS reported in **Table 2** might have various competing explanation (accounting for GxE interaction and marginal exposure effect, two-stage vs one-stage approach, winner’s curse, etc). We therefore argue that those external GWAS mostly provide a baseline for comparing the relative performance of the four interaction models conducted within CHARGE (*i.e.* for the two smoking and two drinking exposures). We also used those GWAS to investigate the links between marginal and interaction effect, and observed differences when repeating the analysis using in-sample marginal effect. While previous work demonstrated that the latter solution is valid (18), there are argument for using either strategy. Finally, we used 1000 Genomes Project(19) data as a reference for deriving LD between SNPs. Here we simply merged available data from the corresponding ancestries, however, finer approaches such as ancestry sub-sampling matching the proportion of each ancestry in CHARGE might be used in future studies.

# Supplementary Figures

## Figure S1 QQplots

Q-Q plots of the p-values from the 1df test of interaction (a-d) and from the 2df joint test (e-h) performed on the four blood pressure traits (DBP, SBP, PP, MAP) and the three lipid traits (HDL, LDL, TG) for the four exposure: current drinking (a, e), drinking habits (b, f), current smoking (c, g), and ever smoking (d, h). Because of highly significant results observed with the 2df test (e-h), we zoomed on *P*-values above 10^-20^ ($-{log}_{10}\left( P \right)=20$) for panels a-d to better visualize the adequacy between the observed and expected p-values distribution for null variants.


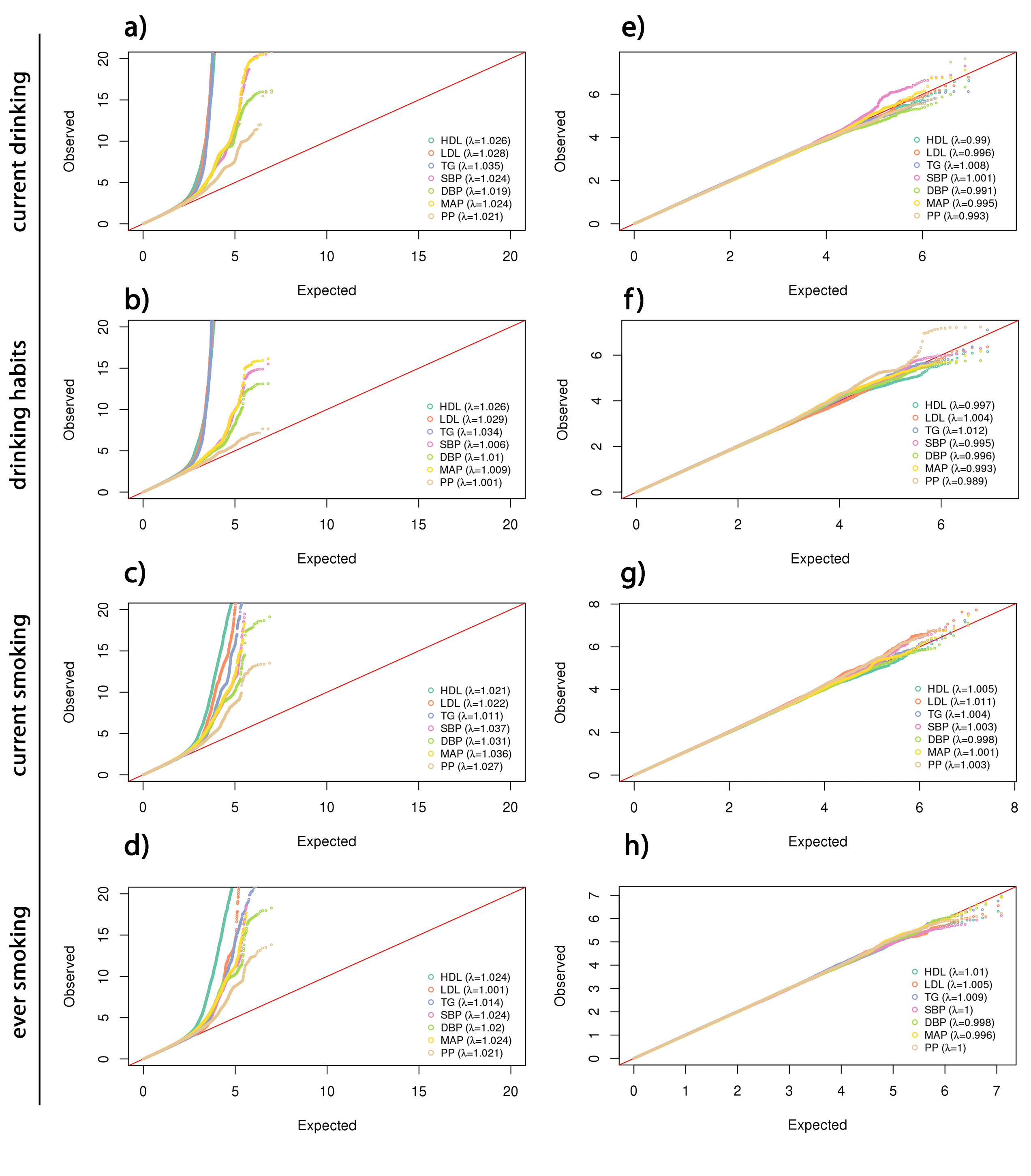


## Figure S2. Proportion of exposed individuals

We plotted the proportion (in %) of exposed individuals for the three lipids traits (HDL, LDL, TG) and four blood pressure traits (DBP, SBP, MAP and PP) in each ancestry separately: European ancestry (EUR), African ancestry (AFR), Asian ancestry (ASA), Hispanic ancestry (HA) and trans-ancestry (ALL_POP). Panel a) shows the mean in the combined analysis with point sizes proportional to the sample size. Panel b) shows the distribution across cohorts meta-analyzed at stage 1 and 2.


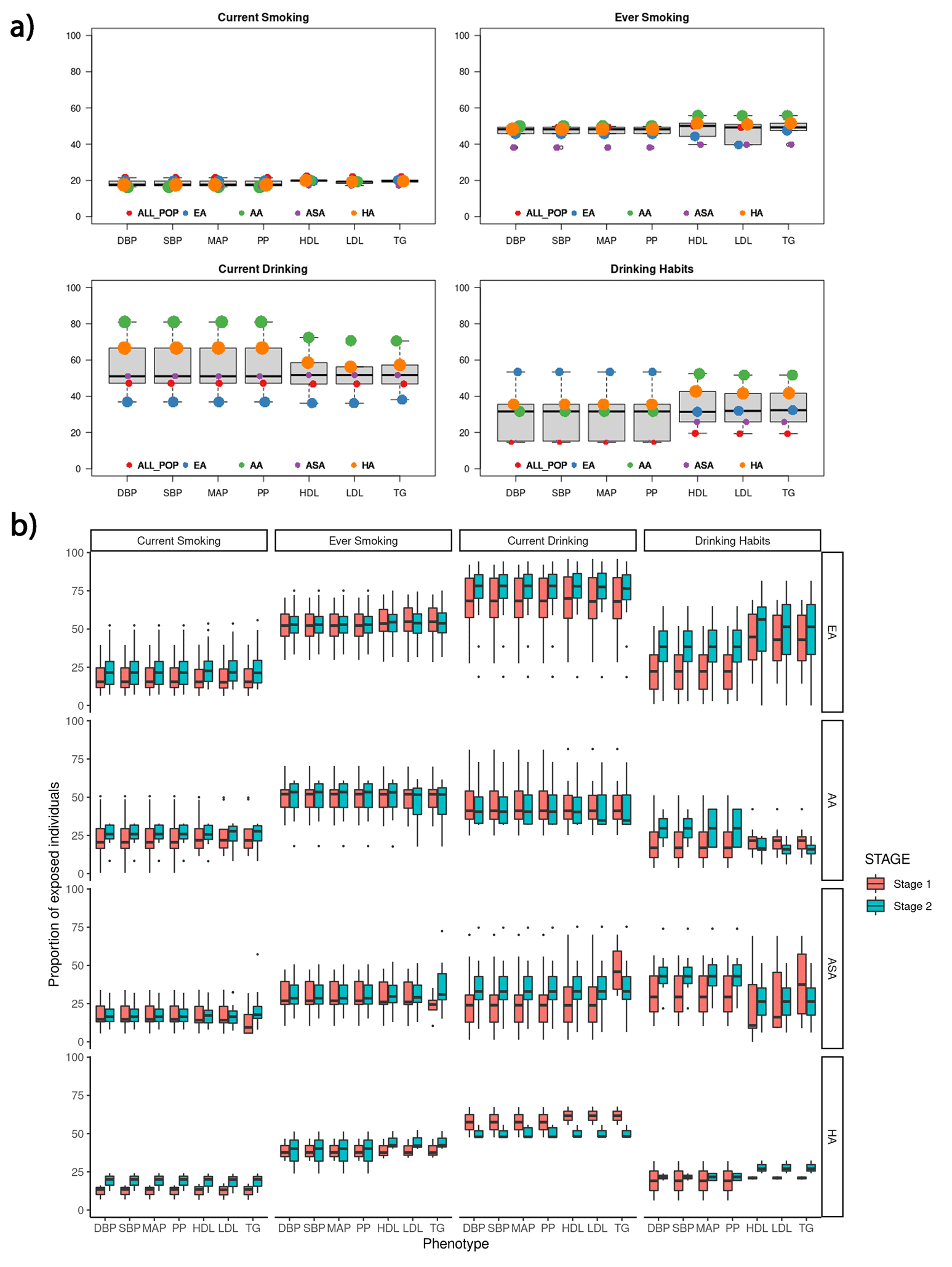


## Figure S3. Distribution of independent signals per locus

We derived the distribution of the number of independent association signal per locus after clumping using a LD threshold of 0.2 for the 2df joint test in the trans-ancestry analysis (ALL_POP), and each ancestry: European (EA), Asian (ASA), African (AA), and Hispanic (HA) ancestries. Distribution were derived after merging results from all exposure-phenotypes pairs. Blue bars indicate the count of loci for each number of independent signals. Red lines show the cumulative number of loci.


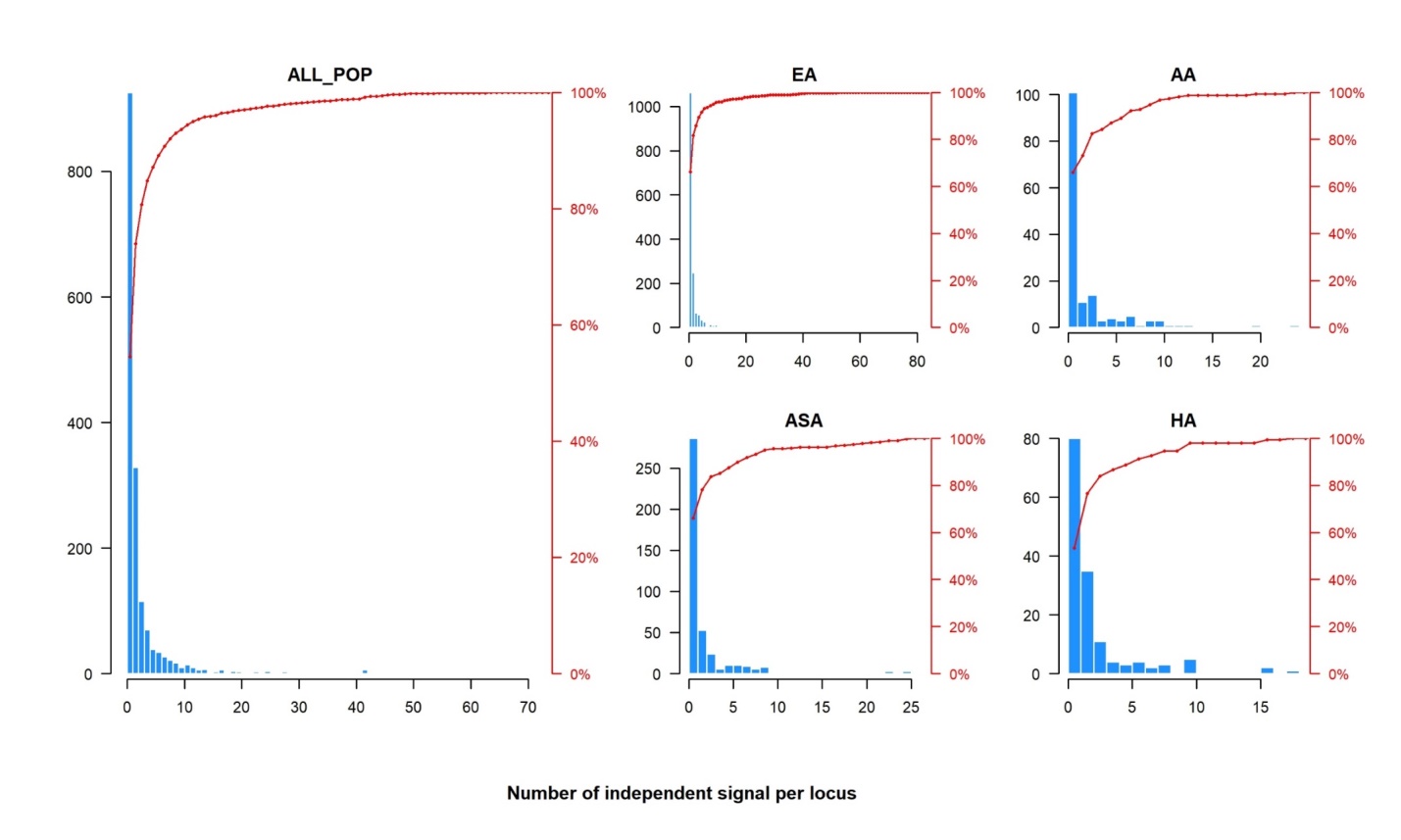


## Figure S4. Comparison of the 2df joint test in CHARGE against external marginal effect GWAS

We plotted the -log10(p-value) of the lead SNPs per associated loci for the 2df joint test in CHARGE obtain after combining stage 1&2 against external marginal effect GWAS. Overlapping loci are in black, loci specific to the CHARGE are in blue, and loci specific to external GWAS are in red. The overlapping loci show strong correlation of -log10_pval_ (r^2^=0.75). As showed in the inner barplot displaying the median -log10(p-value) for the three categories, most of the non-overlapping loci display smaller signal as compared to the overlapping ones.


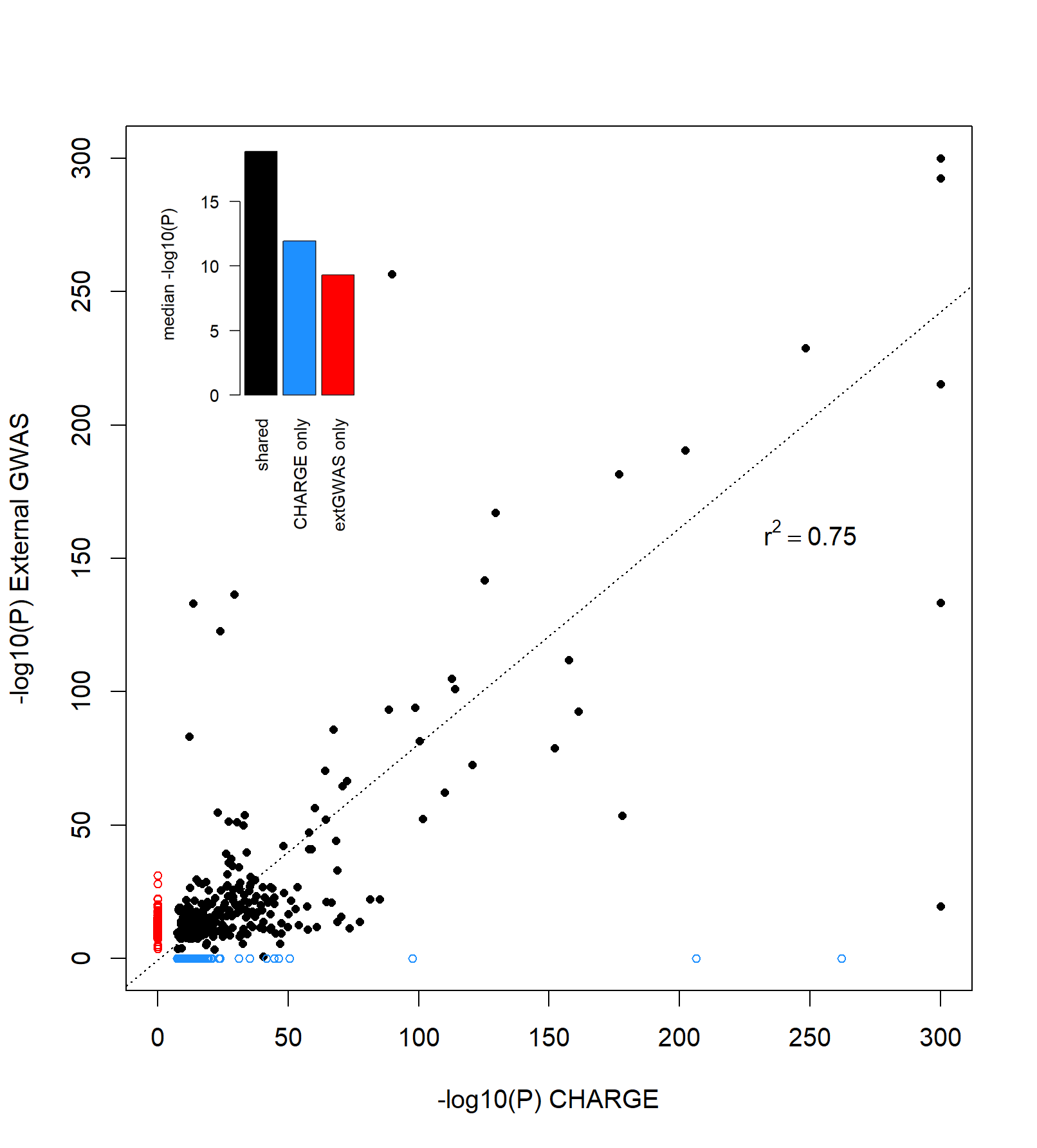


## Figure S5. Comparison of HDL results

We compared the identified loci for HDL across the four exposures. Panel (**a**) shows the 2df chi-squared at loci identified by the SNP by current drinking interaction GWAS (x axis) that were also identified by the other exposures GWAS (y axis). For those loci, the signal is, as expected, highly correlated ($\rho$=0.99, P=6.2x10^-23^, $\rho$=1.00, *P*=2.2x10^-68^, $\rho$=0.99x10^-22^) –i.e. the four analyses have been conducted on the same outcome for almost the same participants. The only difference is the exposure used in the interaction effect. Panel (**b**) shows the observed chi-squared from the 1DF interaction meta-analysis for the 108, 103, 67, and 69 loci found by the current drinking, drinking habits, current smoking and ever smoking exposures, respectively. Blue bars correspond to the mean chi-squared per exposure and equal 1.57 (*P*= 8.7x10^-5^), 1.58 (*P*= 1.2x10^-4^), 1.07 (*P*=0.32) and 1.09 (*P*=0.28), where *P*-value were derived empirically. In brief, for each exposure we simulated 1M replicates including each the same number of chi-square (e.g. 108 for current drinking, etc) draw under the null, and derived for each replicate the mean chi-square. The P-value was derived as the proportion of replicates with a mean larger than the observed one.


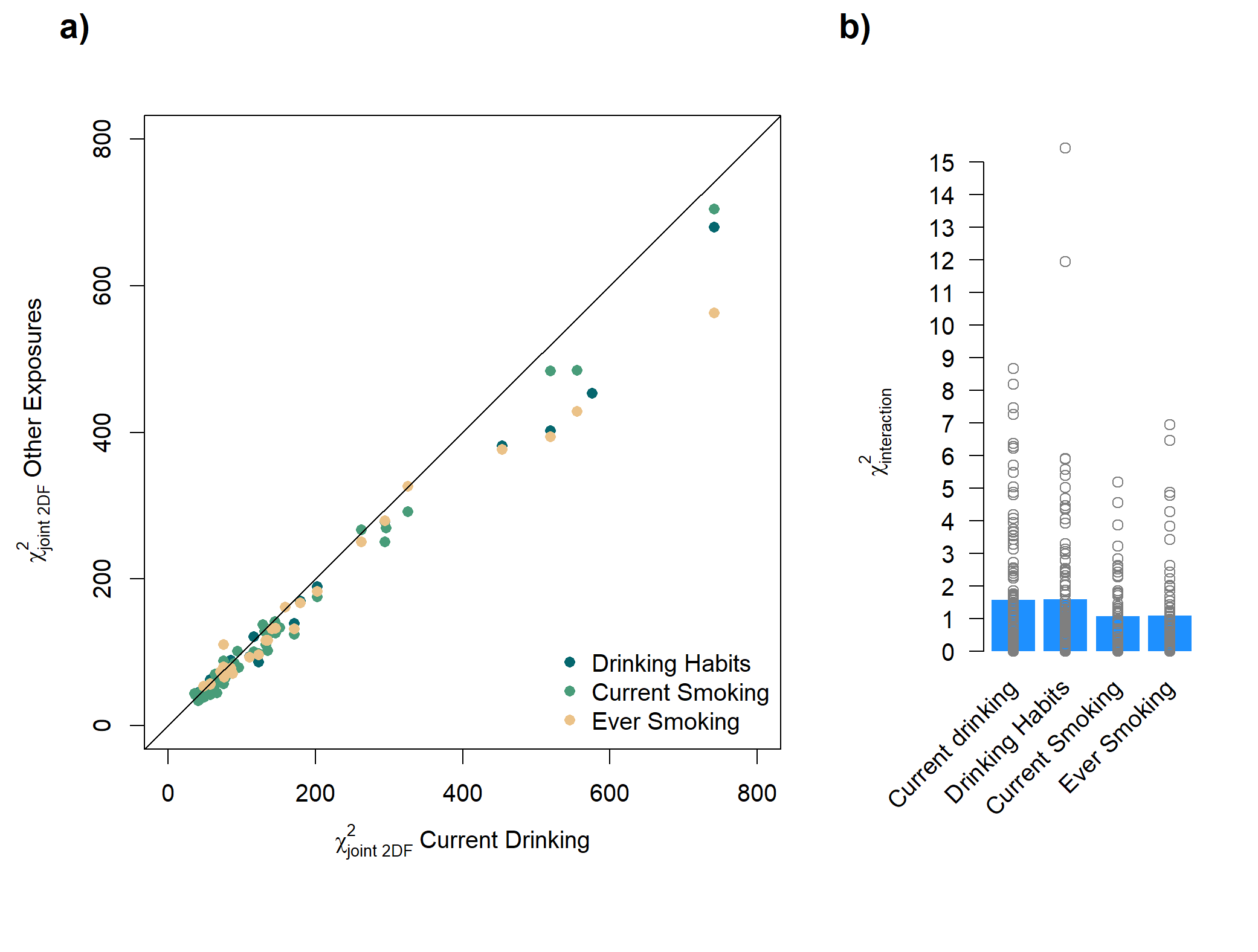


## Figure S6. Marginal and interaction effects among SNPs significant with the 2df test

For each phenotype-exposure-ancestry combination, we extracted the independent SNPs found associated with the 2df test. We then derived the number of SNPs for which marginal and interaction coefficients were concordant (both positive or both negative, grey bar) and discordant (orange + red bar). The former pattern indicates enhanced genetic effect among exposed individuals, while the latter indicates decreased genetic effect among exposed individuals. Across SNPs with discordant marginal and interaction effect, the red bars show the few cases where the discordant interaction effect is large enough to induce an opposite genetic effect between exposed and unexposed. Nominally significant differences are indicated by one star. Those passing a stringent Bonferroni correction are indicated with two stars.


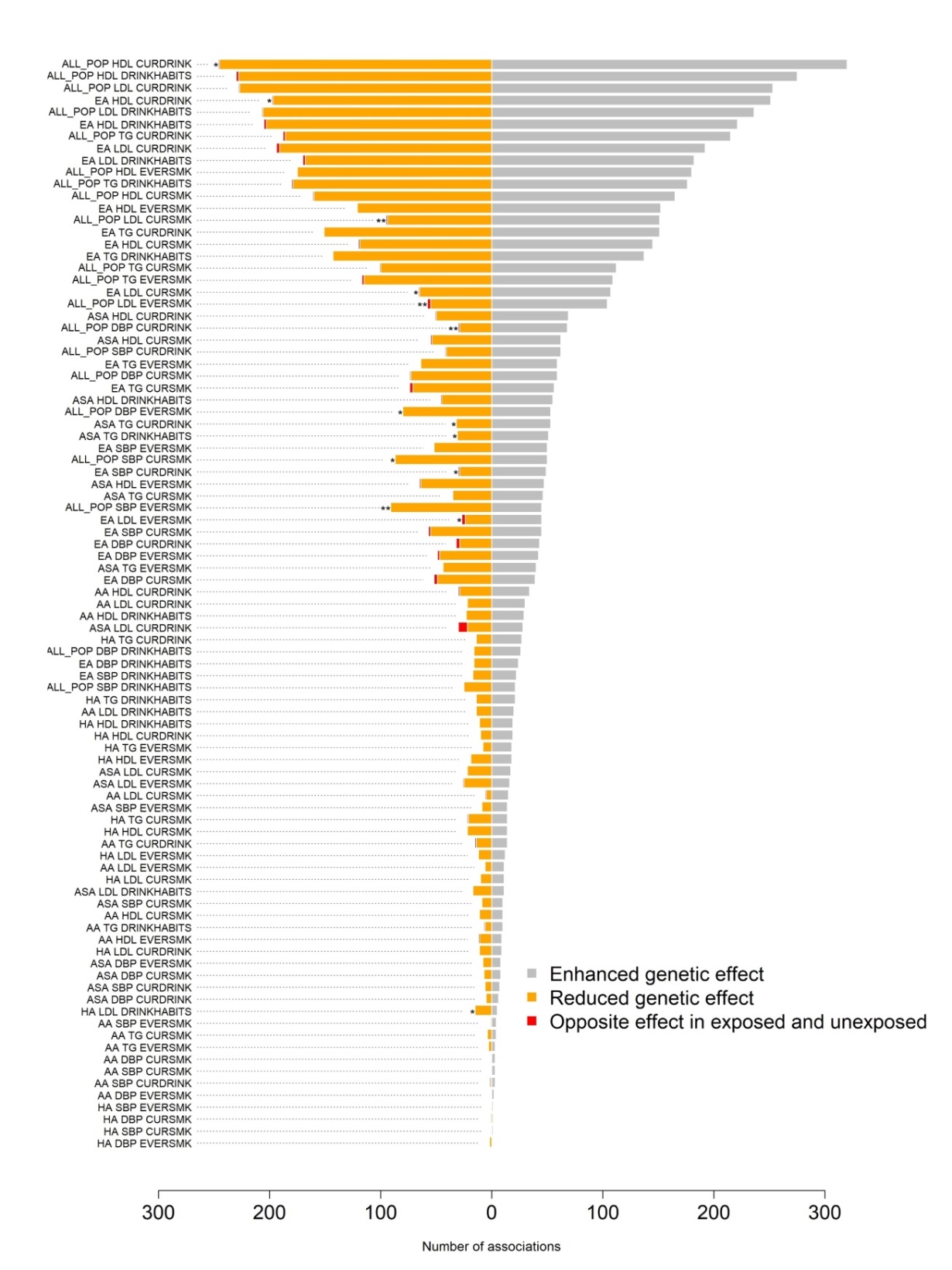


## Figure S7. Interaction effect at previously reported loci

Forest plots of single SNP interaction Z-scores at independent genome-wide significant SNPs retrieved from previously published large-scale GWAS on blood pressure traits (DBP, SBP, PP) and lipid traits (HDL, LDL, TG). For each SNP, we re-derived Z-score so that the coded allele is the allele associated with increasing phenotypic values in the previous marginal GWAS. Z-scores for interaction were plotted separately for current drinking (A), drinking habits (B), current smoking (C), and ever smoking (D), along the Z-scores for the marginal model (E).

**
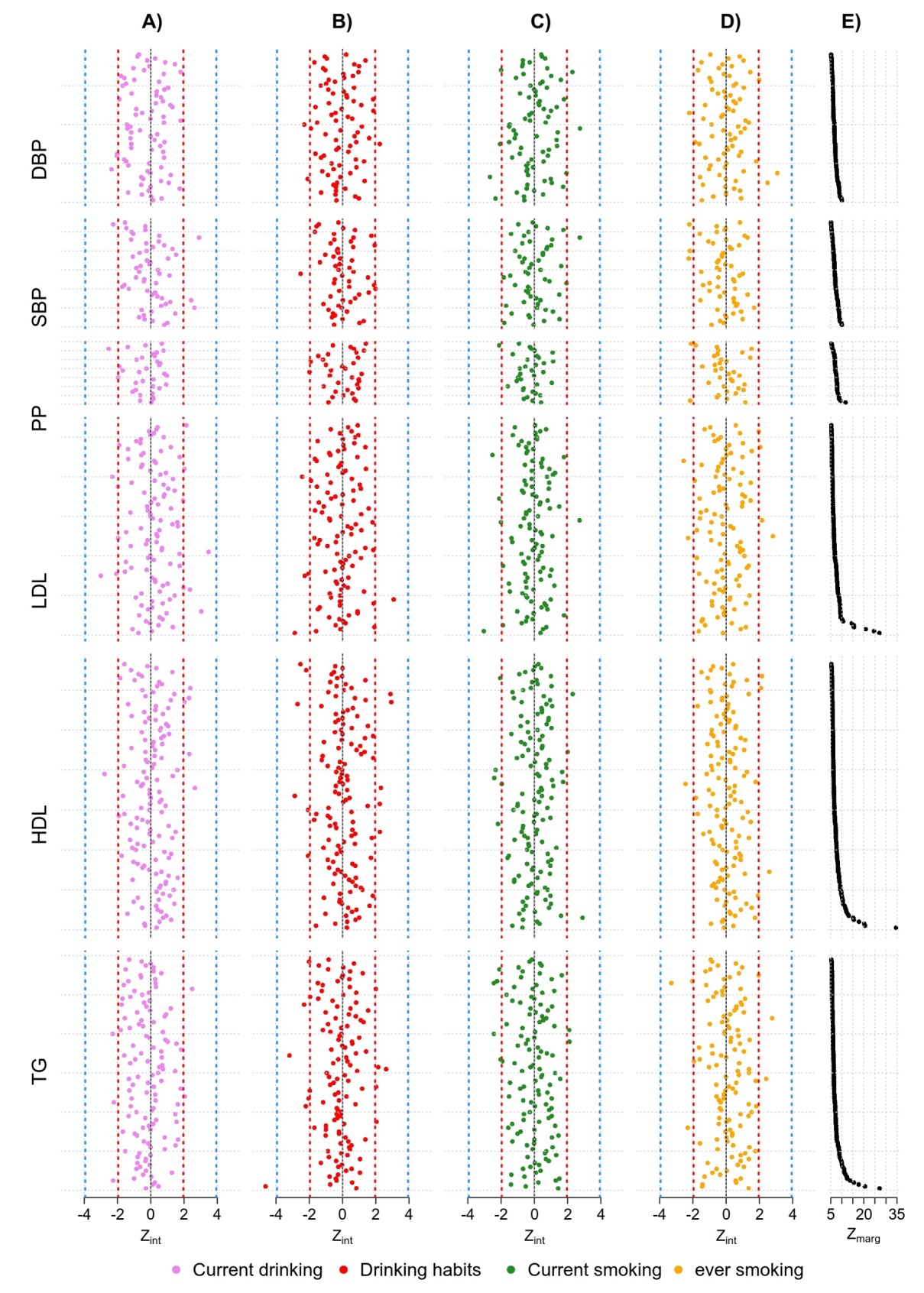
**

## Figure S8. Example of power for 2 df interaction test

For a range of GxE interaction effect sizes, we plotted the statistical power to detect such an interaction effect using the standard 1-df GxE test (blue), 2-df joint test (green), and the 2-step procedure screening (red). In the 2 step procedure, candidate SNPs are selected based on their marginal genetic effect (step 1) and then tested for an interaction effect (step 2). Power estimation were computed assuming a GWAS of 1 million SNPs (type I error rate of 5x10^-8^) with an allele frequency qA=0.25 and including N=10,000 subjects. In these computations,the exposure is assumed to be binary with a prevalence pE=0.4. We also assumed the absence of genetic and environmental marginal effects (Bg=Be=0) in an additive genetic model.The population standard deviation of the phenotype Y was equal to 1. Calculations were performed using the software package Quanto+(20).


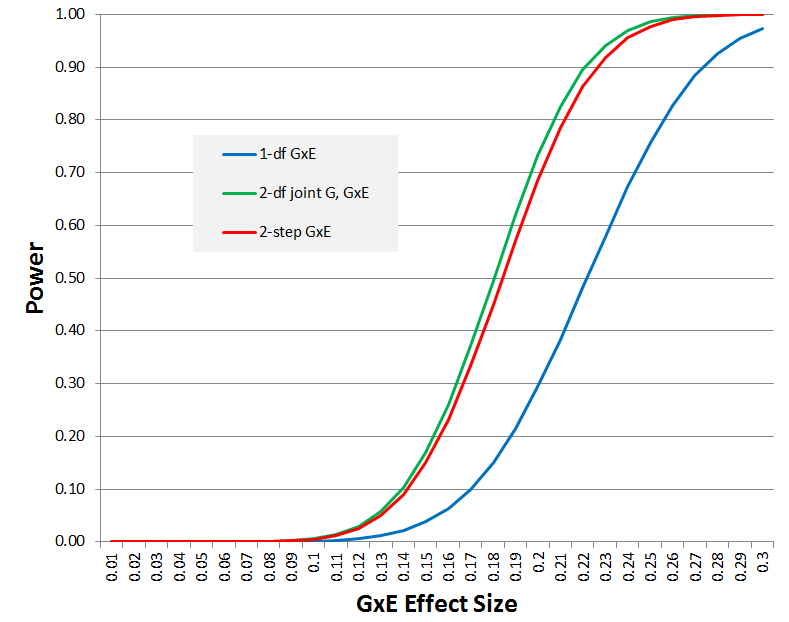


## Figure S9. Potential power for 2-step approach

We plotted for each environmental exposure, current drinking (a), drinking habits (b), current smoking (c) and ever smoking (d), the proportion of independent SNPs displaying an interaction *p*-value (P_int_) below 0.05 in CHARGE across bins of variants selected from the marginal genetic effect observed at stage 1 in the CHARGE data. Those bins were defined as sets of independent variants with *p*-value for marginal genetic effect (P_marg_) lower than a given threshold (x axis). Each of the five phenotypes are represented by a plain color line. All analyses used stage 1 1df interaction results from European ancestry individuals. Under the null hypothesis of no correlation, the proportion follows a binomial distribution with parameter 0.05 (the black dashed line), independent of the threshold for P_marg_. Grey areas indicate the Wilson score confidence interval for an alpha threshold of 0.05 (dark grey) and 1 x 10^-4^ (light grey).


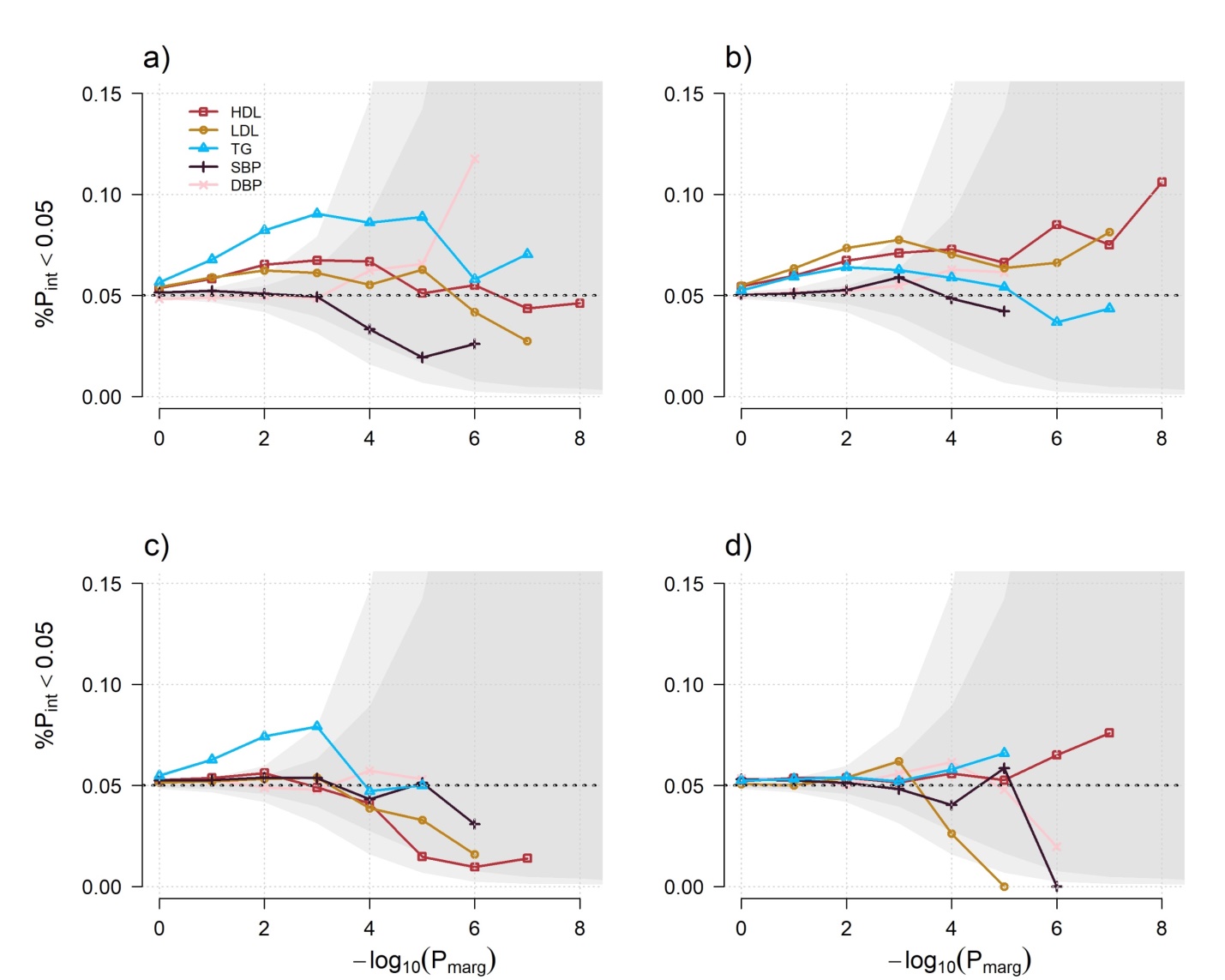


## Figure S10. Variability in heritability estimation

We estimated the genetic heritability of each phenotype in exposed and unexposed individuals separately using the LD Score (LDSC), while filtering out an increasing number of variants based on their p-value for heterogeneity in the meta-analysis (P>0, >0.3, >0.5, >0.8, >0.9). Panel (a) shows the estimated heritabilities as a function of the ratio of the LDSC intercept minus 1 over the mean chi-square minus 1. Panel (b) shows the estimated heritabilities as a function of the number of SNPs used for the estimation. Panel (c) shows the heritability per P-value threshold for each phenotype-exposure status pair. Among those, the baseline heritability estimated without filtering (i.e. for P>0) is indicated by a green circle, and the selected heritability (with the lowest ratio and a number of SNPs larger than 200K) is indicated by a red dot.


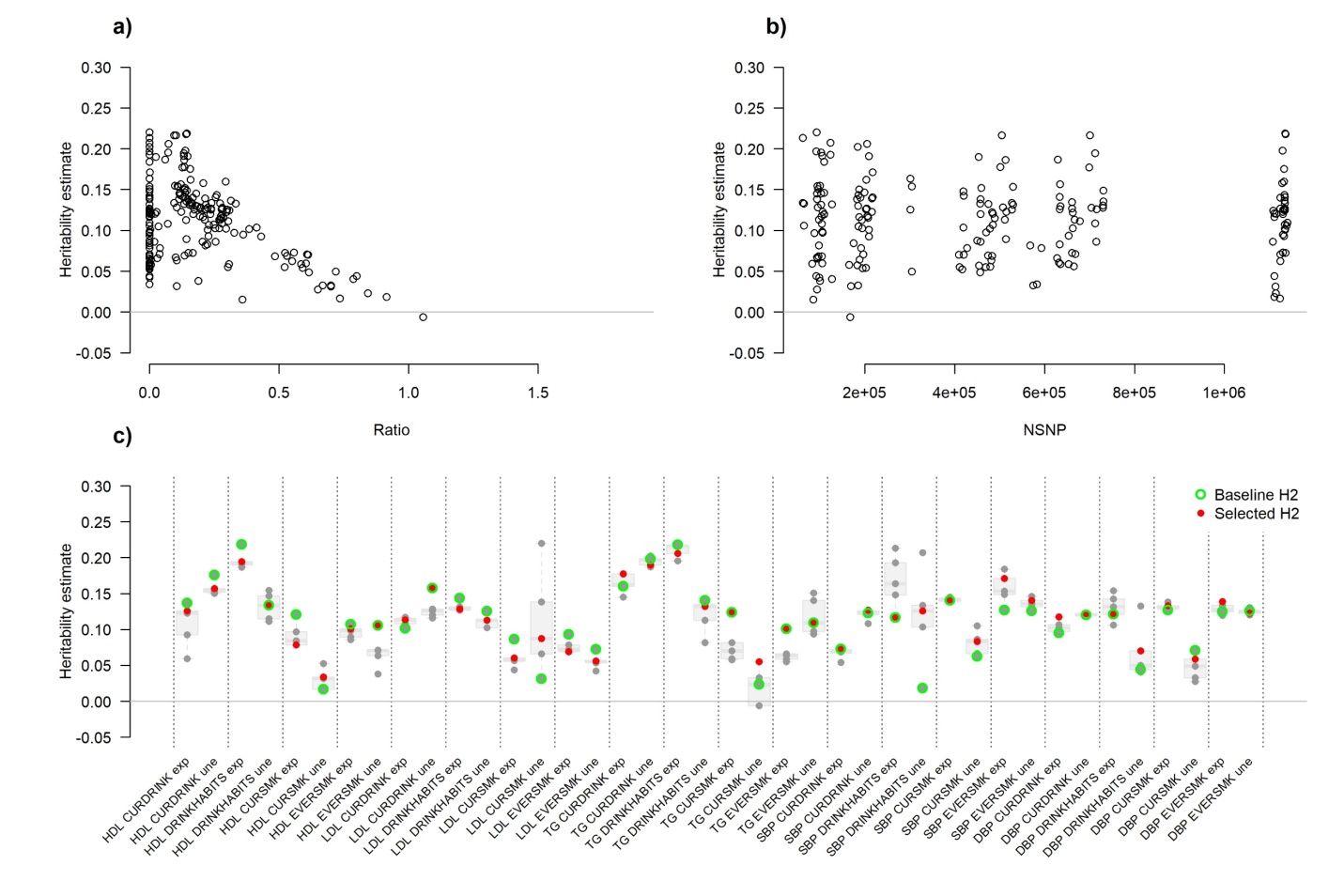


## Figure S11. Annotation enrichment for DBP

Partitioned heritability for DBP in European ancestry individuals. Summary statistics from combined and stratified analyses (“Marginal”: combined exposure groups for current smoking (largest sample on average), "+": exposed, "-": unexposed) were used. Color indicates enrichment of heritability. Red stripped squares indicate an enrichment above 20. The size of the squares are proportional to –log10(P). All annotations significant after correction for the 185 tests performed (P < 0.00027) are indicated by the "*" sign. For clarity, we only showed annotations significant in at least one exposure stratum.


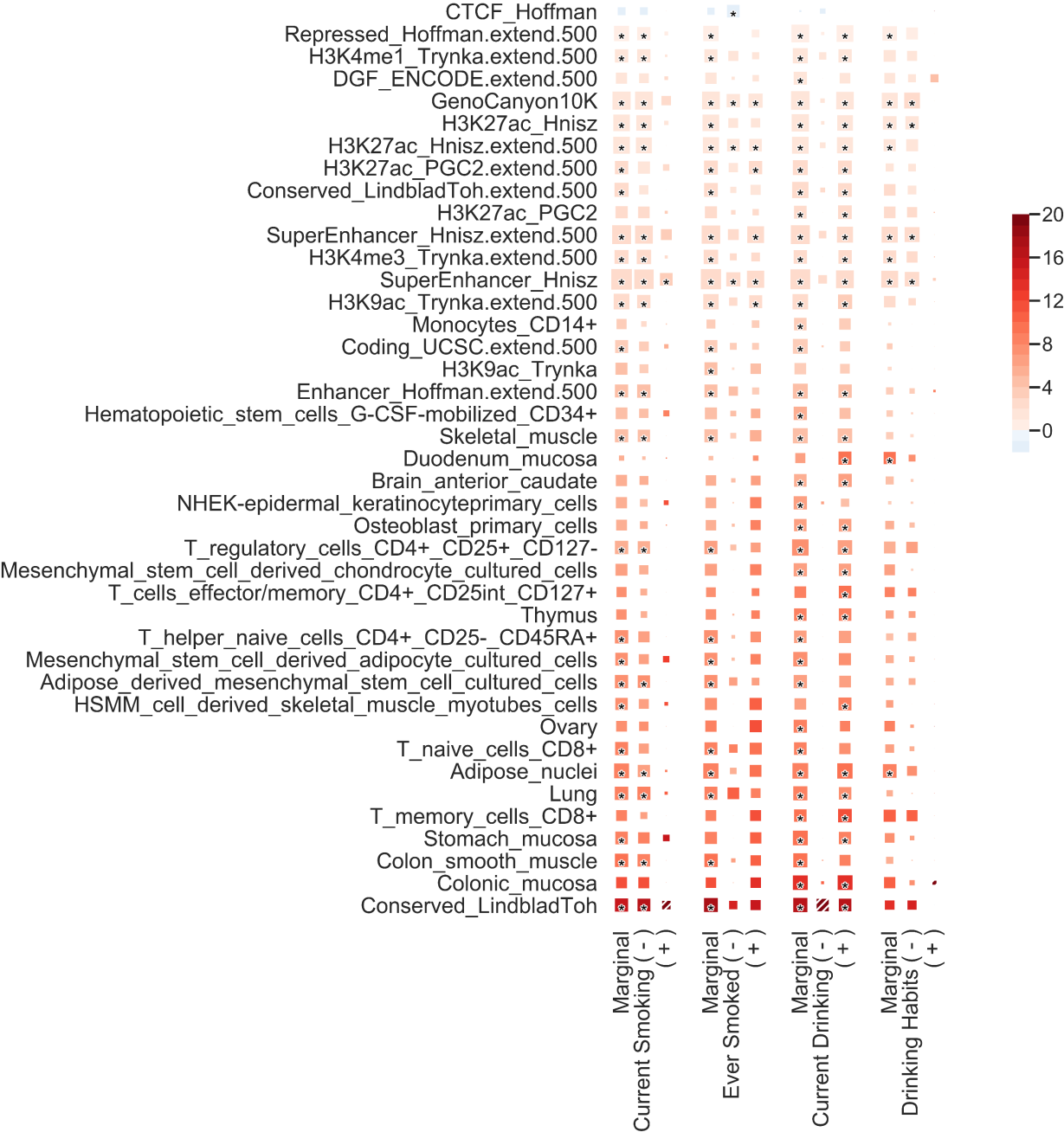


## Figure S12. Annotation enrichment for SBP

Partitioned heritability for SBP in European ancestry individuals. Summary statistics from combined and stratified analyses (“Marginal”: combined exposure groups for current smoking (largest sample on average), "+": exposed, "-": unexposed) were used. Color indicates enrichment of heritability. Red stripped squares indicate an enrichment above 20. The size of the squares are proportional to –log10(P). All annotations significant after correction for the 185 tests performed (P < 0.00027) are indicated by the "*" sign. For clarity, we only showed annotations significant in at least one exposure stratum.


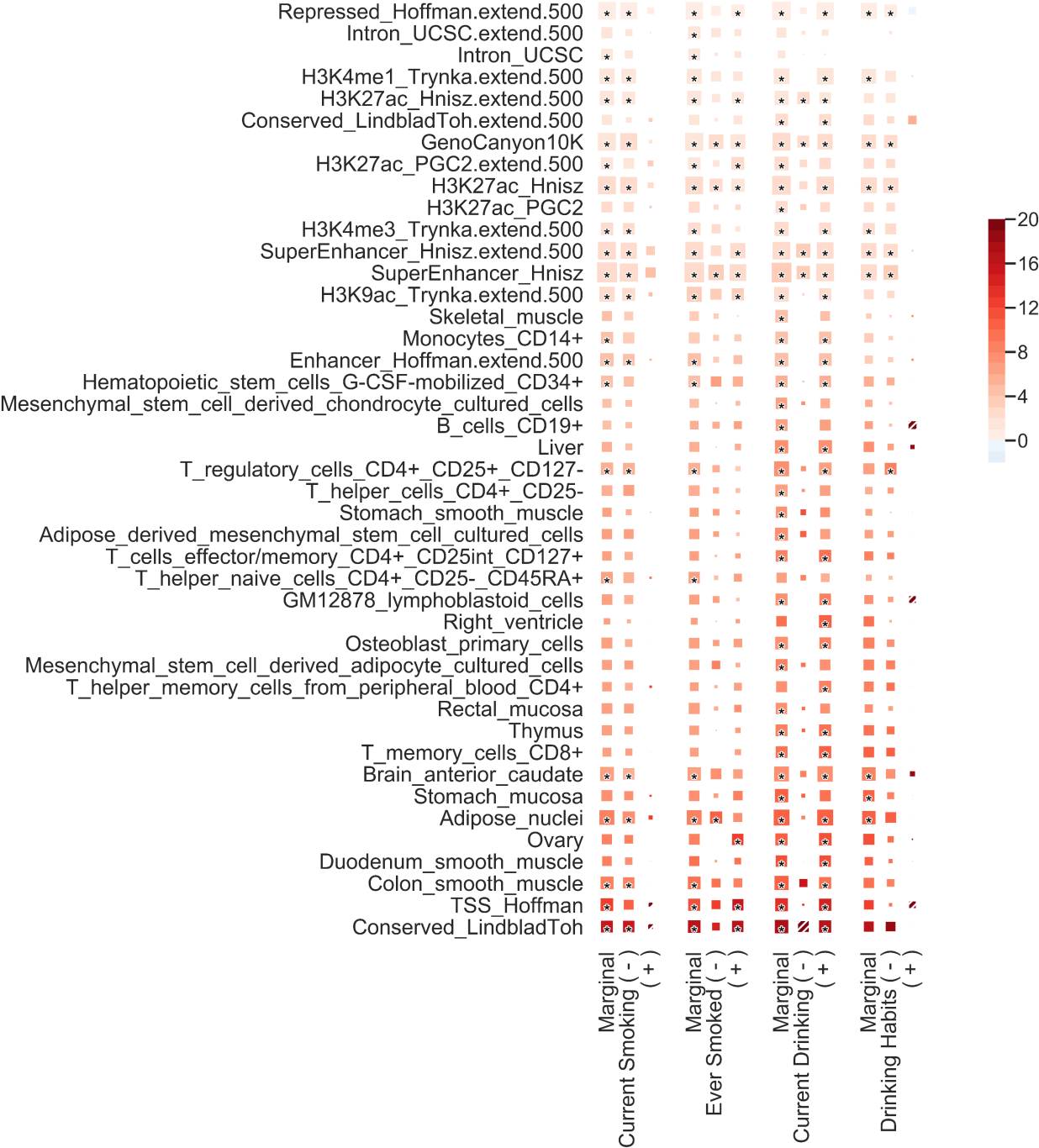


## Figure S13. Annotation enrichment for HDL

Partitioned heritability for HDL in European ancestry individuals. Summary statistics from combined and stratified analyses (“Marginal”: combined exposure groups for current smoking (largest sample on average), "+": exposed, "-": unexposed) were used. Color indicates enrichment of heritability. Red stripped squares indicate an enrichment above 20. The size of the squares are proportional to –log10(P). All annotations significant after correction for the 185 tests performed (P < 0.00027) are indicated by the "*" sign. For clarity, we only showed annotations significant in at least one exposure stratum.


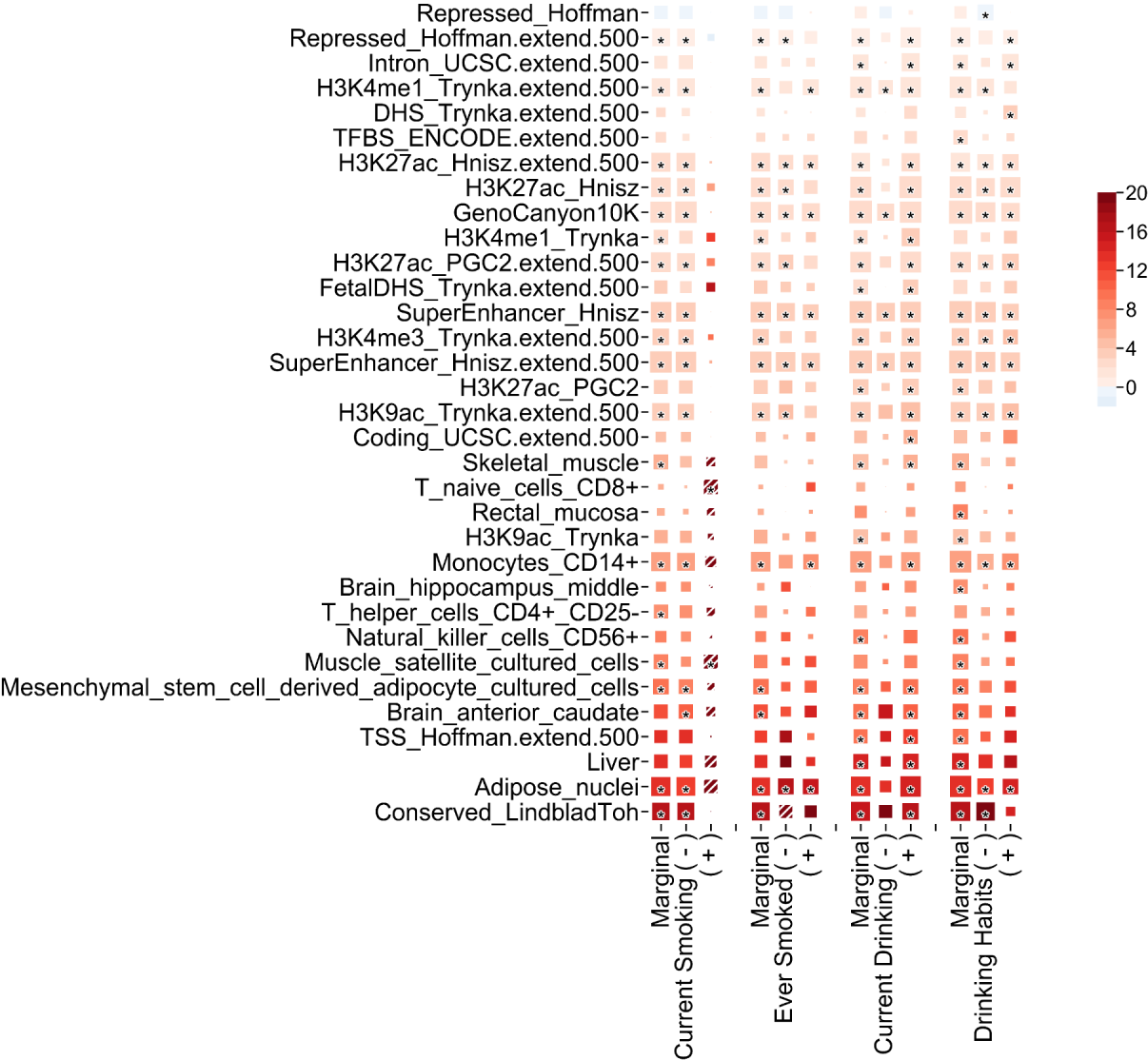


## Figure S14. Annotation enrichment for LDL

Partitioned heritability for LDL in European ancestry individuals. Summary statistics from combined and stratified analyses (“Marginal”: combined exposure groups for current smoking (largest sample on average), "+": exposed, "-": unexposed) were used. Color indicates enrichment of heritability. Red stripped squares indicate an enrichment above 20. The size of the squares are proportional to –log10(P). All annotations significant after correction for the 185 tests performed (P < 0.00027) are indicated by the "*" sign. For clarity, we only showed annotations significant in at least one exposure stratum.


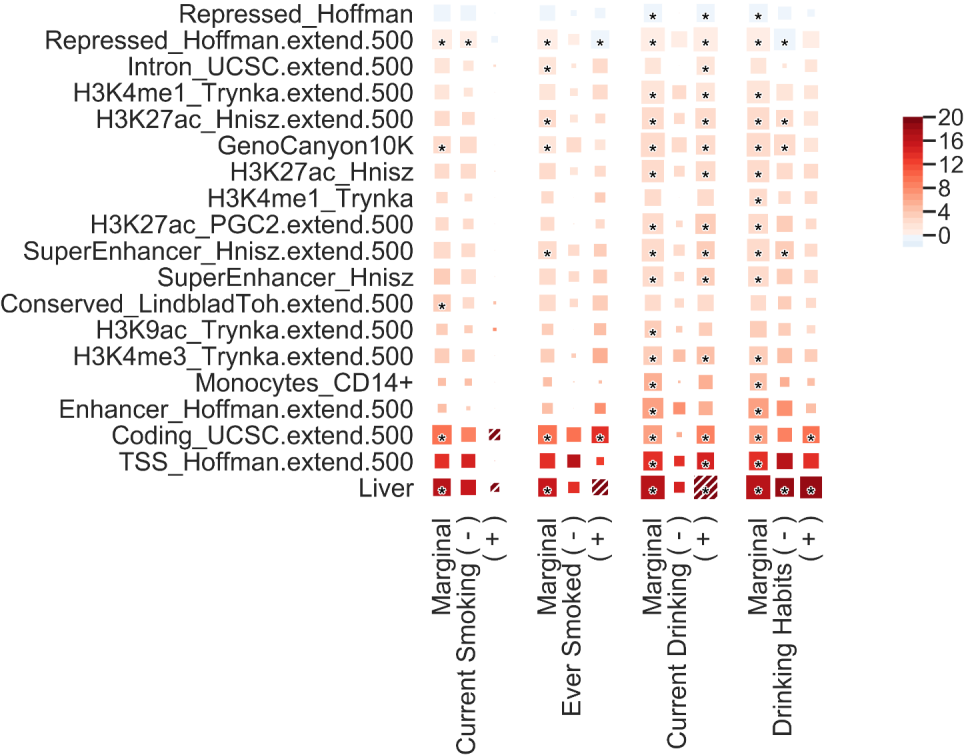


## Figure S15. Annotation enrichment for TG

Partitioned heritability for TG in European ancestry individuals. Summary statistics from combined and stratified analyses (“Marginal”: combined exposure groups for current smoking (largest sample on average), "+": exposed, "-": unexposed) were used. Color indicates enrichment of heritability. Red stripped squares indicate an enrichment above 20. The size of the squares are proportional to –log10(P). All annotations significant after correction for the 185 tests performed (P < 0.00027) are indicated by the "*" sign. For clarity, we only showed annotations significant in at least one exposure stratum.


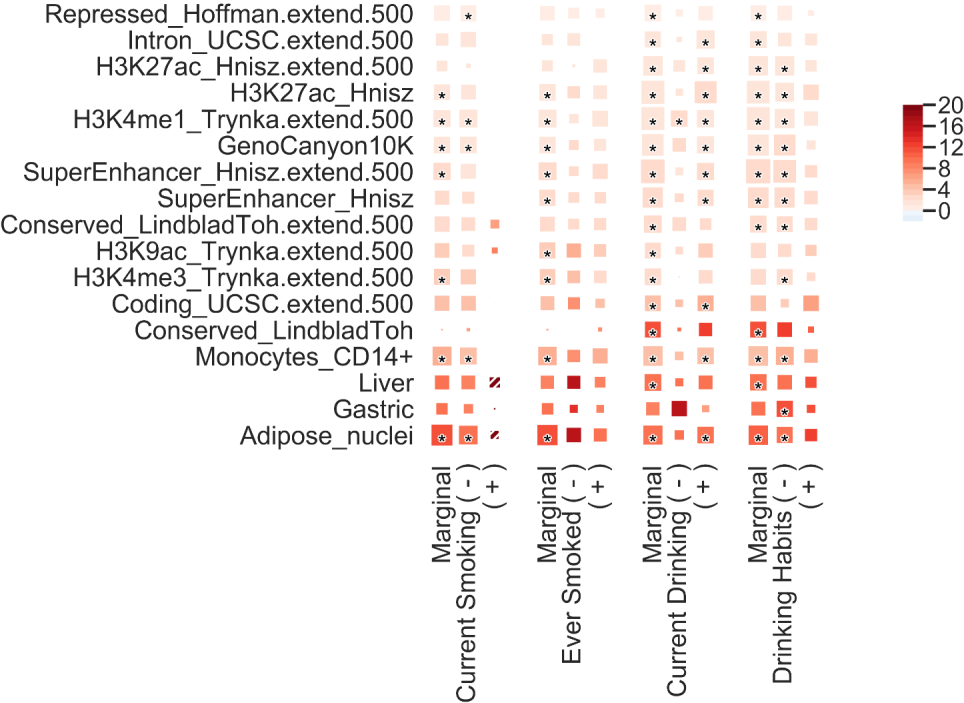


## Figure S16. . Functional enrichment across baseline and GenoSkyline+ annotations

Partitioned heritability for each lipid-exposure in European ancestry individuals across 180 functional annotations. Enrichment among unexposed individuals (X axis) is plotted against enrichment among exposed individuals (Y axis). The gradient of red and size of each data point indicates respectively enrichment and significance in the whole sample (including all exposed and all unexposed individuals). For clarity, we only included data points with enrichment’s standard error smaller than the maximum observed in the whole sample in either exposed or unexposed strata. Such large variability mostly reflects poor estimation due to small sample size, making interpretation unreliable. Several outcome-exposure combinations, such as HDL-drinking, HDL-ever smoking, and BP-ever smoking displays highly consistent enrichment by exposure. Conversely, LDL-current drinking, LDL-ever smoking, and DBP-current drinking show substantial differences in annotation enrichment.


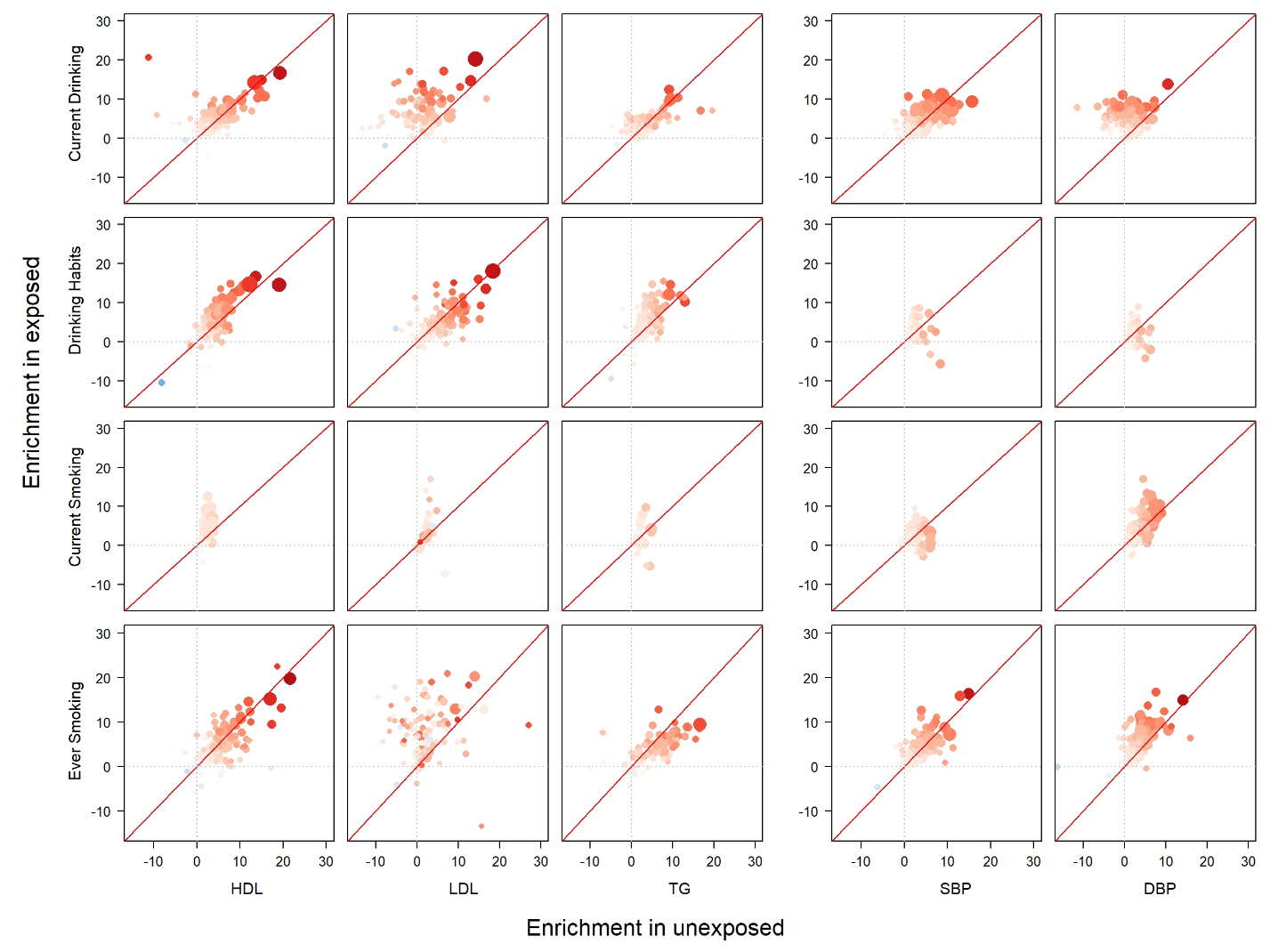


## Figure S17. Cell-type enrichment for DBP

We partitioned the genetic heritability of DBP across different cell-types for each exposure: current drinking (a), current smoking (b), drinking habits (c), and ever smoking (d). Each panel shows the enrichment for the 205 cell-types (y axis), aggregated into tissues (x axis). For each tissue we further derived the overall median enrichment (bold line). Tissues are ordered by the median derived in unexposed individuals.

**
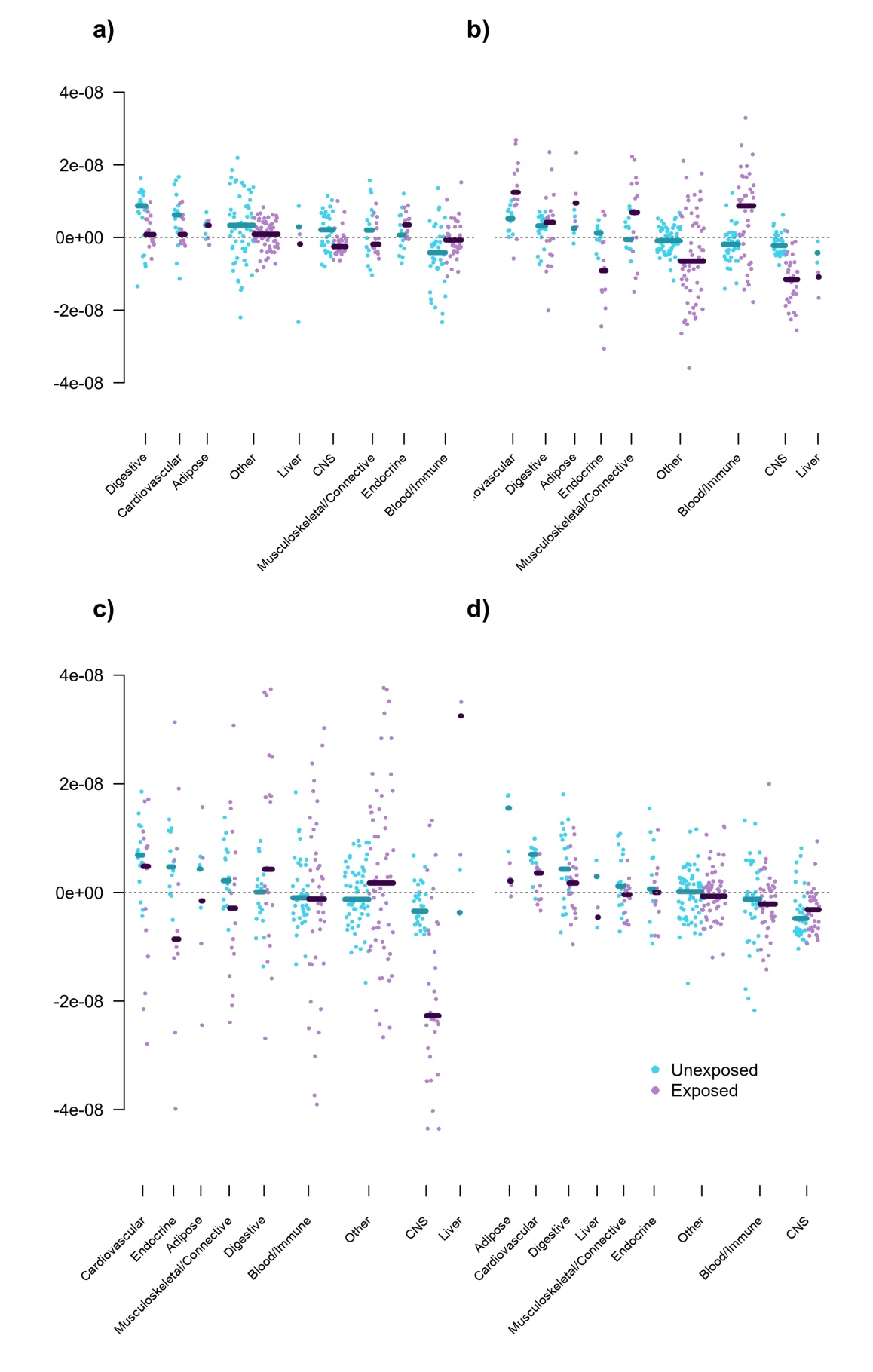
**

## Figure S18. Cell-type enrichment for SBP

We partitioned the genetic heritability of SBP across different cell-types for each exposure: current drinking (a), current smoking (b), drinking habits (c), and ever smoking (d). Each panel shows the enrichment for the 205 cell-types (y axis), aggregated into tissues (x axis). For each tissue we further derived the overall median enrichment (bold line). Tissues are ordered by the median derived in unexposed individuals.


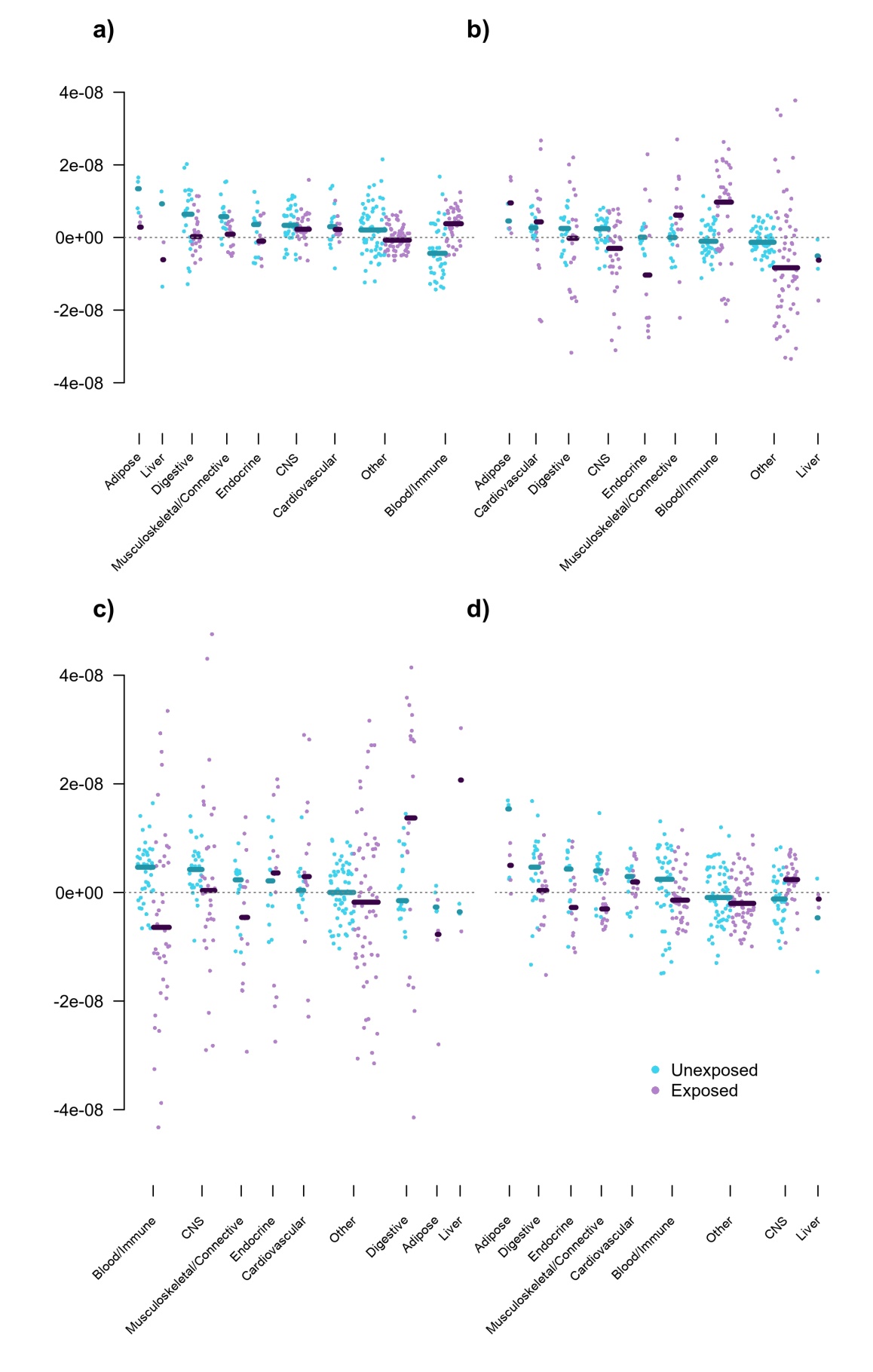


## Figure S19. Cell-type enrichment for HDL

We partitioned the genetic heritability of HDL across different cell-types for each exposure: current drinking (a), current smoking (b), drinking habits (c), and ever smoking (d). Each panel shows the enrichment for the 205 cell-types (y axis), aggregated into tissues (x axis). For each tissue we further derived the overall median enrichment (bold line). Tissues are ordered by the median derived in unexposed individuals.


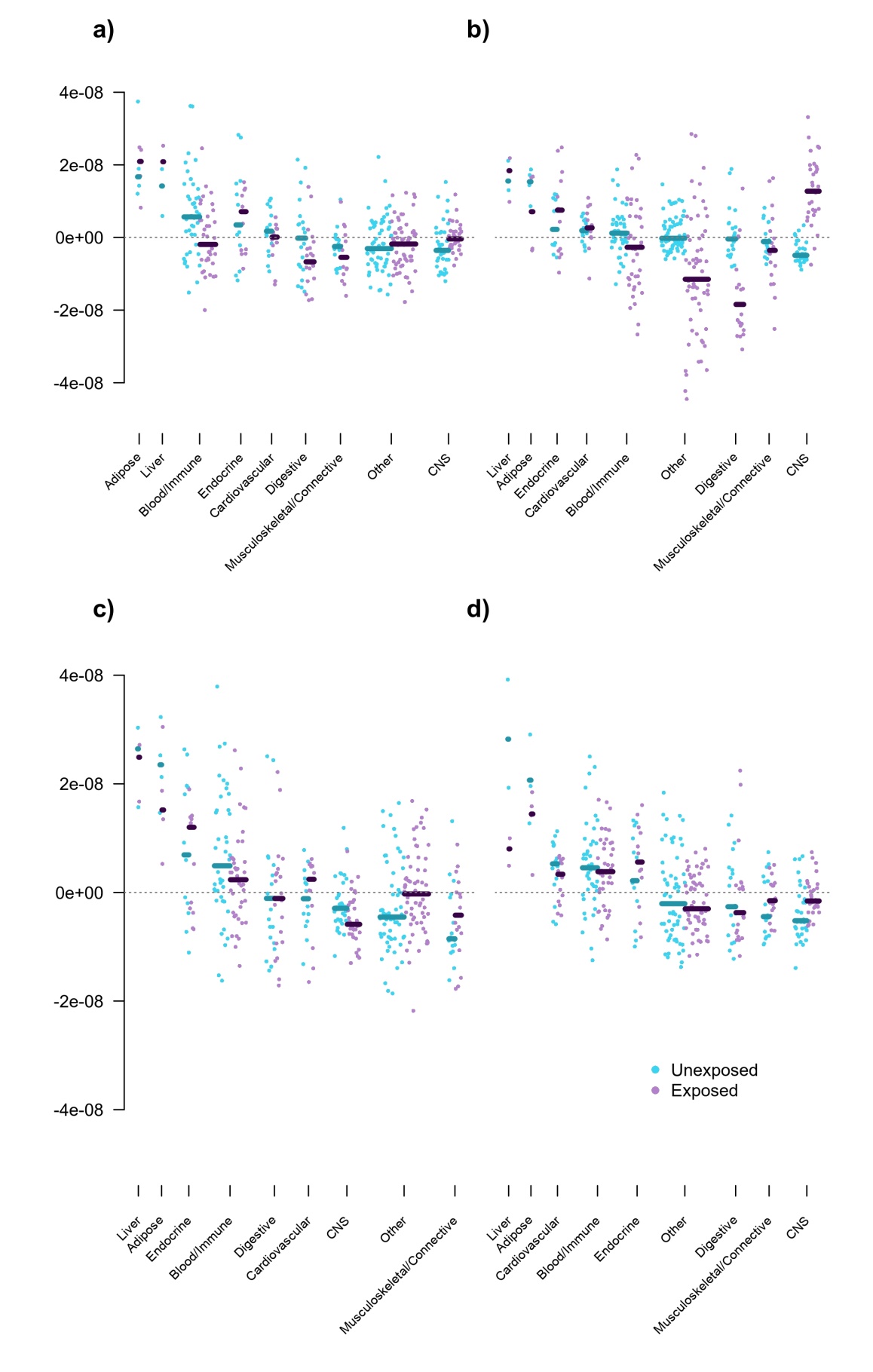


## Figure S20. Cell-type enrichment for LDL

We partitioned the genetic heritability of LDL across different cell-types for each exposure: current drinking (a), current smoking (b), drinking habits (c), and ever smoking (d). Each panel shows the enrichment for the 205 cell-types (y axis), aggregated into tissues (x axis). For each tissue we further derived the overall median enrichment (bold line). Tissues are ordered by the median derived in unexposed individuals.


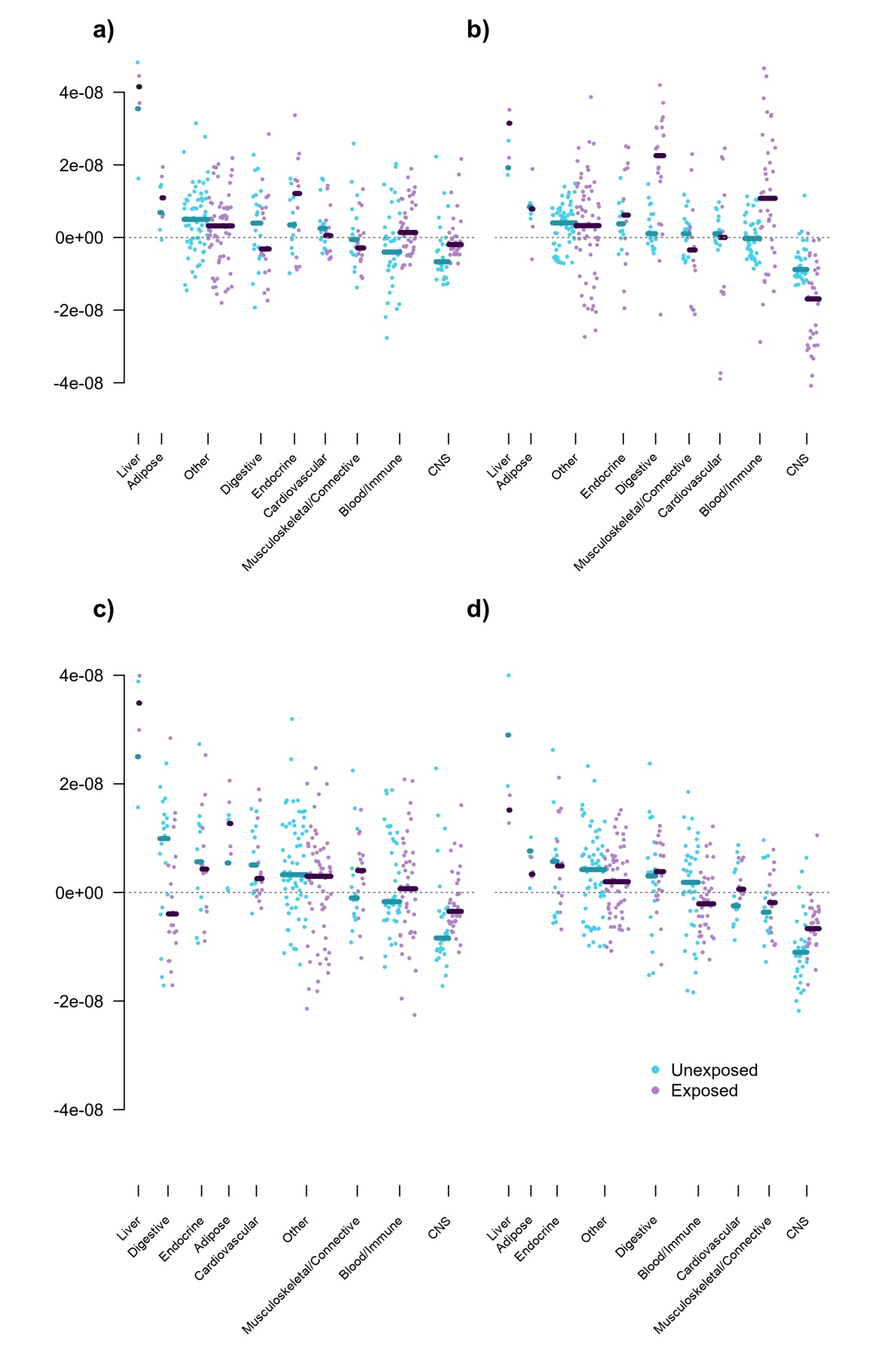


## Figure S21. Cell-type enrichment for TG

We partitioned the genetic heritability of TG across different cell-types for each exposure: current drinking (a), current smoking (b), drinking habits (c), and ever smoking (d). Each panel shows the enrichment for the 205 cell-types (y axis), aggregated into tissues (x axis). For each tissue we further derived the overall median enrichment (bold line). Tissues are ordered by the median derived in unexposed individuals.


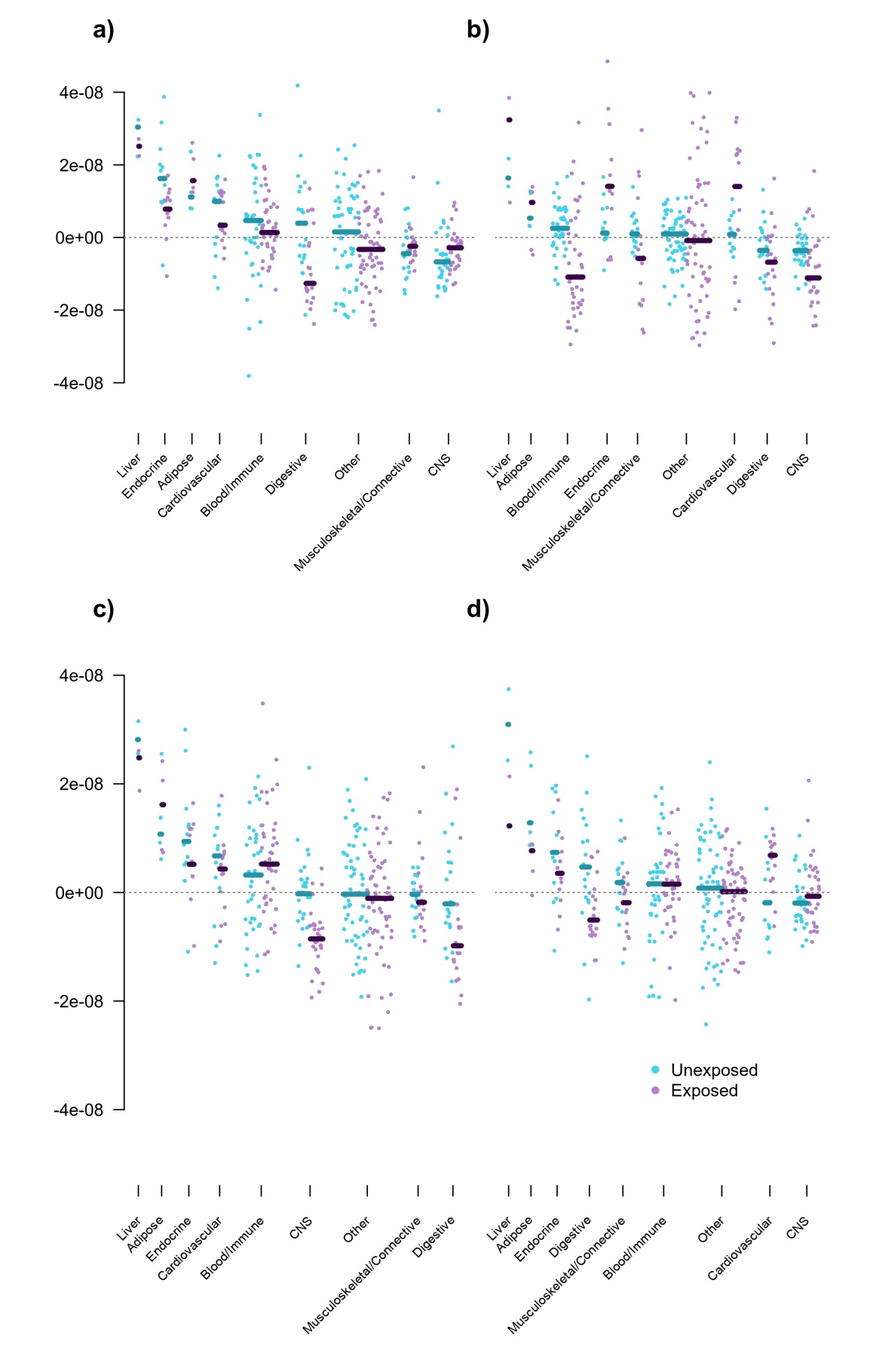


## Figure S22. Example of power for 2 df interaction test

We simulated genotype data in two cohorts of 20,000 individuals under 24 different scenarios to explore the impact of heterogeneity across cohorts (**Table S10**), varying the minor allele frequency, the proportion of exposed individual across cohorts, the presence/absence of main genetic effect, and the presence/absence of interaction effect. We assessed the significance of the interaction effect in each cohort and by performing a meta-analysis using the inverse-variance weighting scheme and the 2 degrees of freedom joint framework. We plotted the *p*-values distribution for the two cohorts and the two methods for each scenario.

**
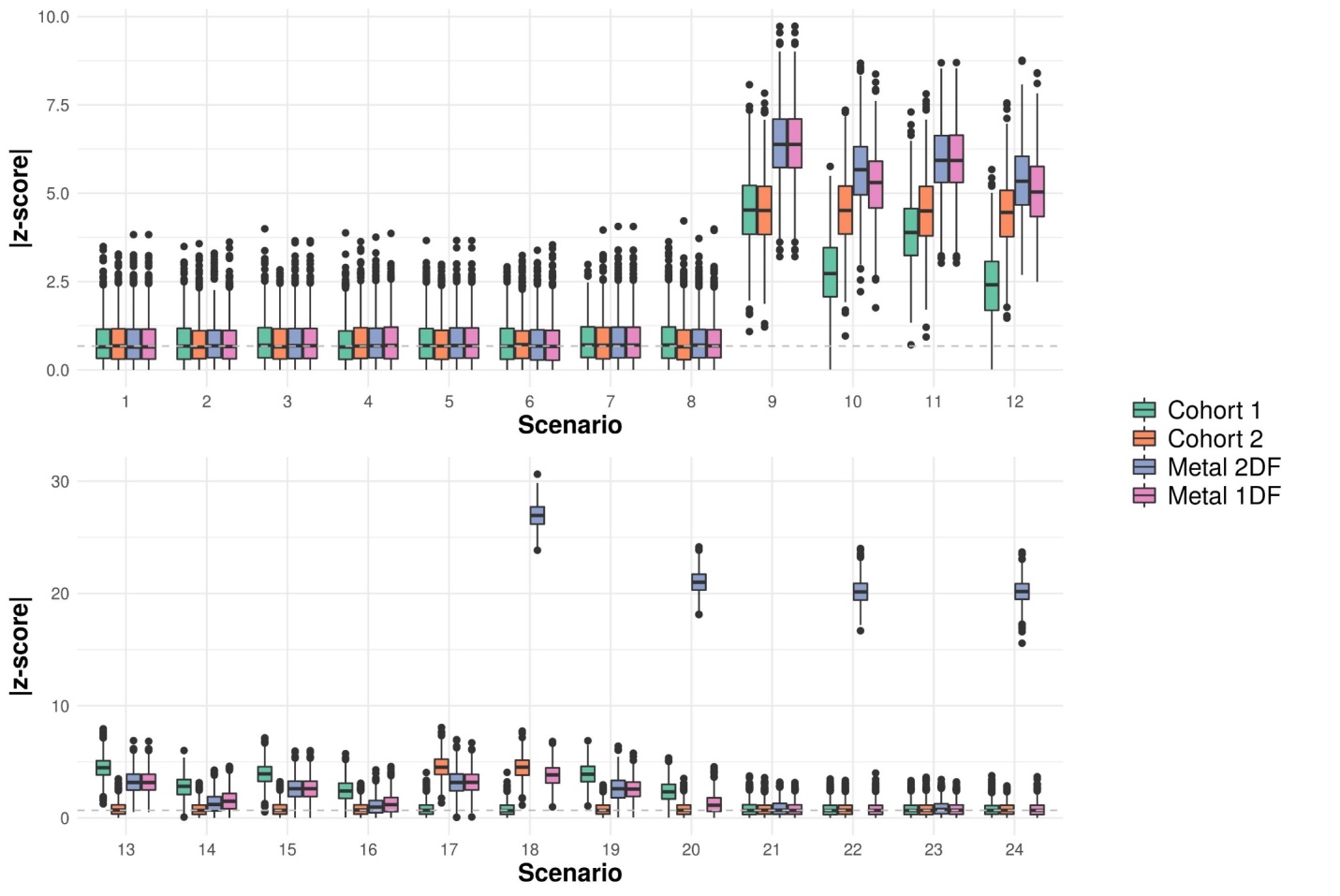
**

## Figure S23. Robustness of the 2df framework for binary exposures

We simulated series of 1,000 replicates, each of them including data on a single genotype (G), a binary exposure (E) and a continuous outcome (Y) for two independent cohorts. For each replicate we performed a linear regression for each cohort using the model Y=E+G+GxE. We then derived the effect estimates and the associated chi-squared of both the main genetic effect and the interaction effect using a standard inverse-variance meta-analysis of the two cohort (1df), and using the 2df framework. We randomly varied all simualtion parameters across replicates and independently in each cohort, including the sample size per cohort (from 5,000 to 15,000), the genotype frequency (from 0.01 to 0.99), the frequency of the exposure, and including or not main and interaction effects in either cohort. Left panel shows the estimated main (*beta_main*) and interaction (*beta_int*) effects for the 1df (X axis) and the 2df (Y axis) approaches, respectively. Right panels show the chi-squared statistics for the corresponding analysis. Data points in red correspond to null model where the generative model does not include main (top panels) or interaction (bottom panels) effects.

**
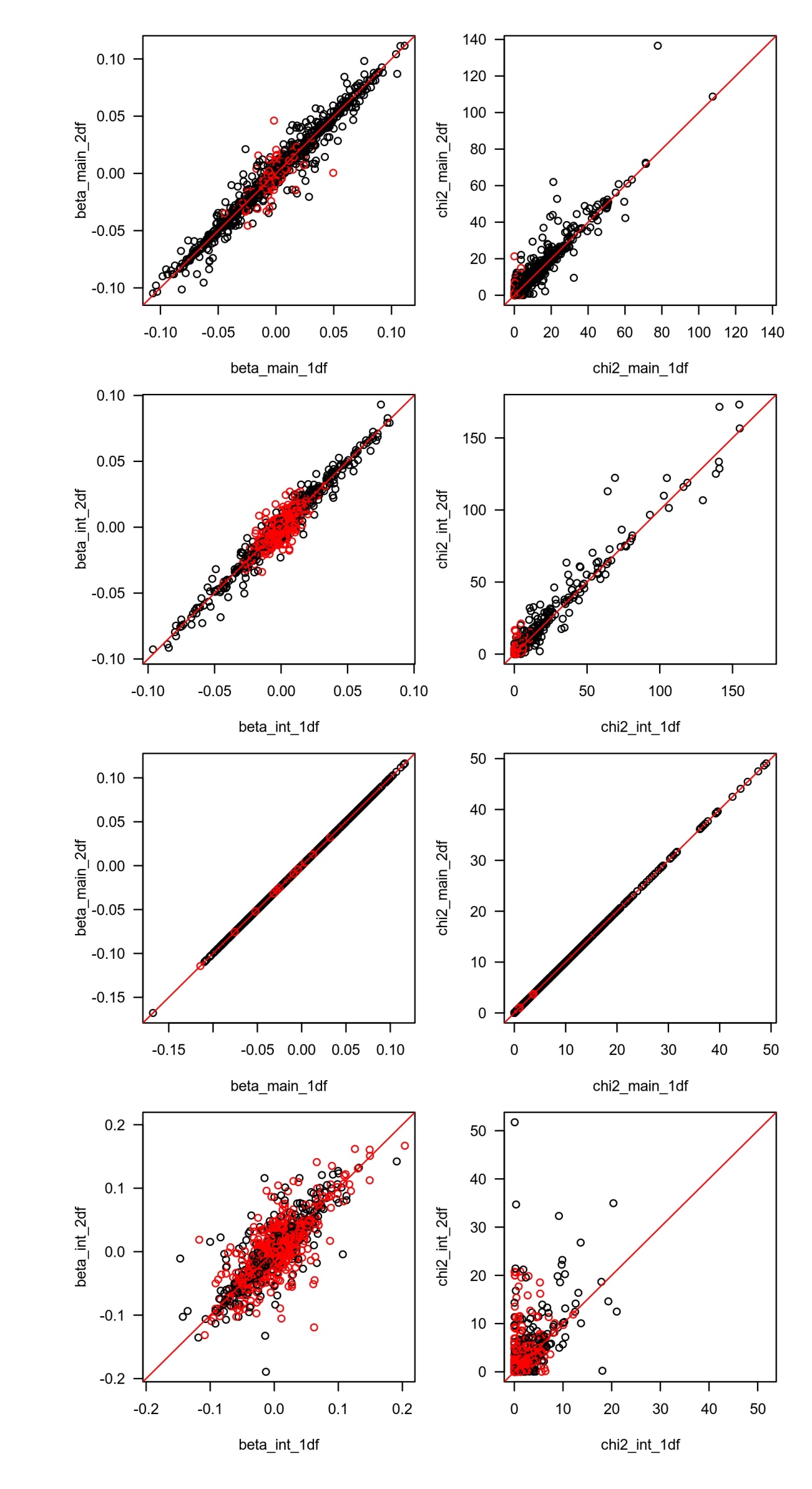
**

## Figure S24. Robustness of the 2df framework

We simulated series of 1,000 replicates, each of them including data on a single genotype (G), a continuous exposure (E) and a continuous outcome (Y) for two independent cohorts. For each replicate we performed a linear regression for each cohort using the model Y=E+G+GxE. We then derived the effect estimates and the associated chi-squared of both the main genetic effect and the interaction effect using a standard inverse-variance meta-analysis of the two cohort (1df), and using the 2df framework. We randomly varied all simualtion parameters across replicates and independently in each cohort, including the sample size per cohort (from 5,000 to 15,000), the genotype frequency (from 0.01 to 0.99), the mean and variance of the exposure, and including or not main and interaction effects in either cohort. Left panel shows the estimated main (*beta_main*) and interaction (*beta_int*) effects for the 1df (X axis) and the 2df (Y axis) approaches, respectively. Right panels show the chi-squared statistics for the corresponding analysis. Data points in red correspond to null model where the generative model does not include main (top panels) or interaction (bottom panels) effects.

**
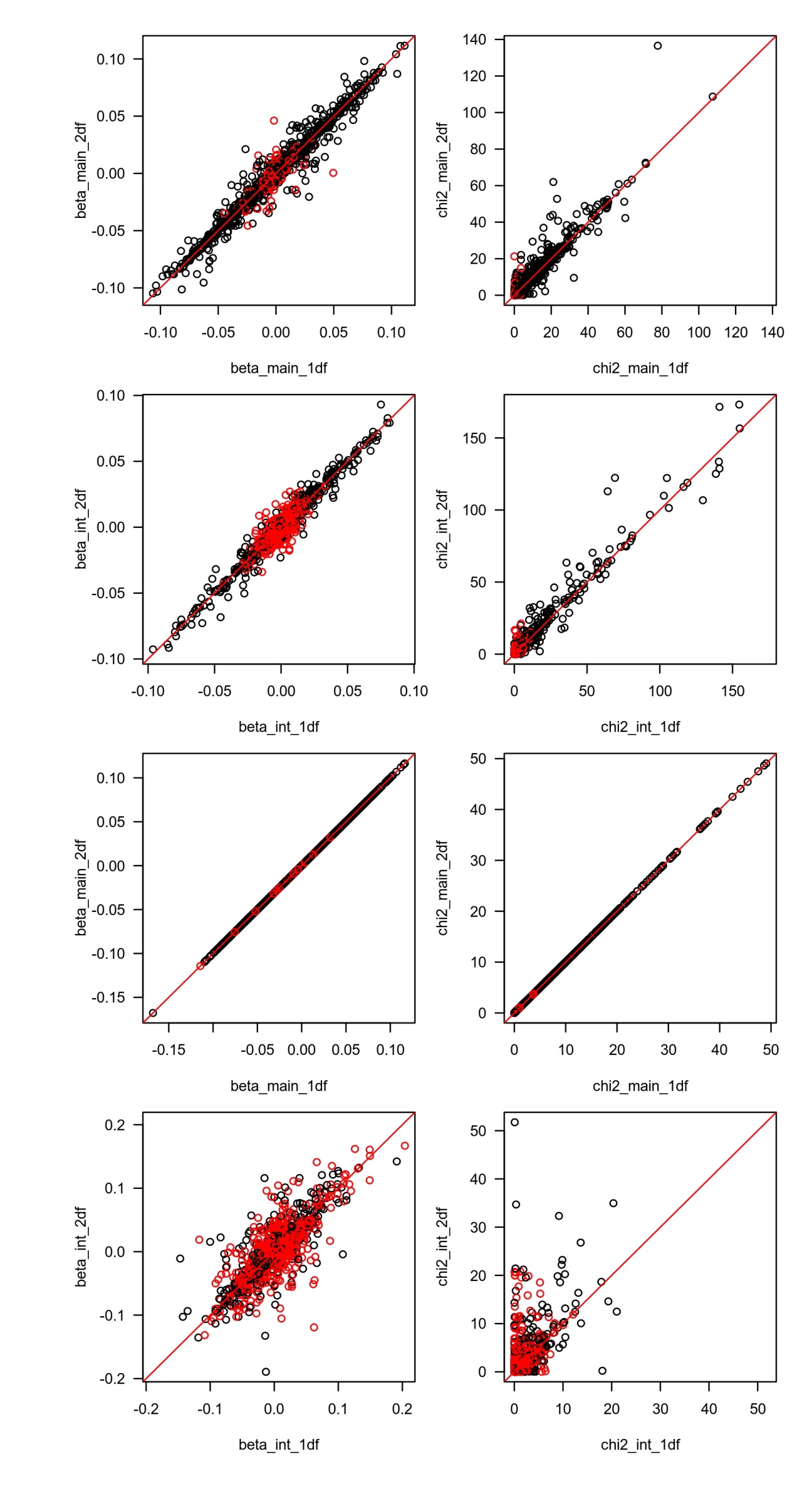
**

## Figure S25. The joint test can capture genetic heterogeneity

We simulated series of 1,000 replicates, each of them including data on a single genotype (G), a binary exposure (E) and a continuous outcome (Y) for two independent cohorts. For each replicate we pooled the two cohorts and performed a linear regression for each cohort using four models: a 1df marginal model only testing the effect of the genetic variants (Y~G), the same model but adjusting for the effect of the exposure (Y~G+E), a joint 2 df model accounting for interaction between G and E, as used in our studies (Y~G+E+GxE), and an alternative joint 2 df accounting for interaction between G and the cohort status (Y~G+C+GxC). The three panels show the QQ plots for the four tests under three generative scenarios. Panel (a) is a null model where there is no genetic effect in neither cohort. Panel (b) is a parsimonuious model where there is homogeneous genetic effect in both cohort. Finally, in panel (c), data were generated assuming heterogeneity between the two cohorts, with some genetic effect only in one cohort.


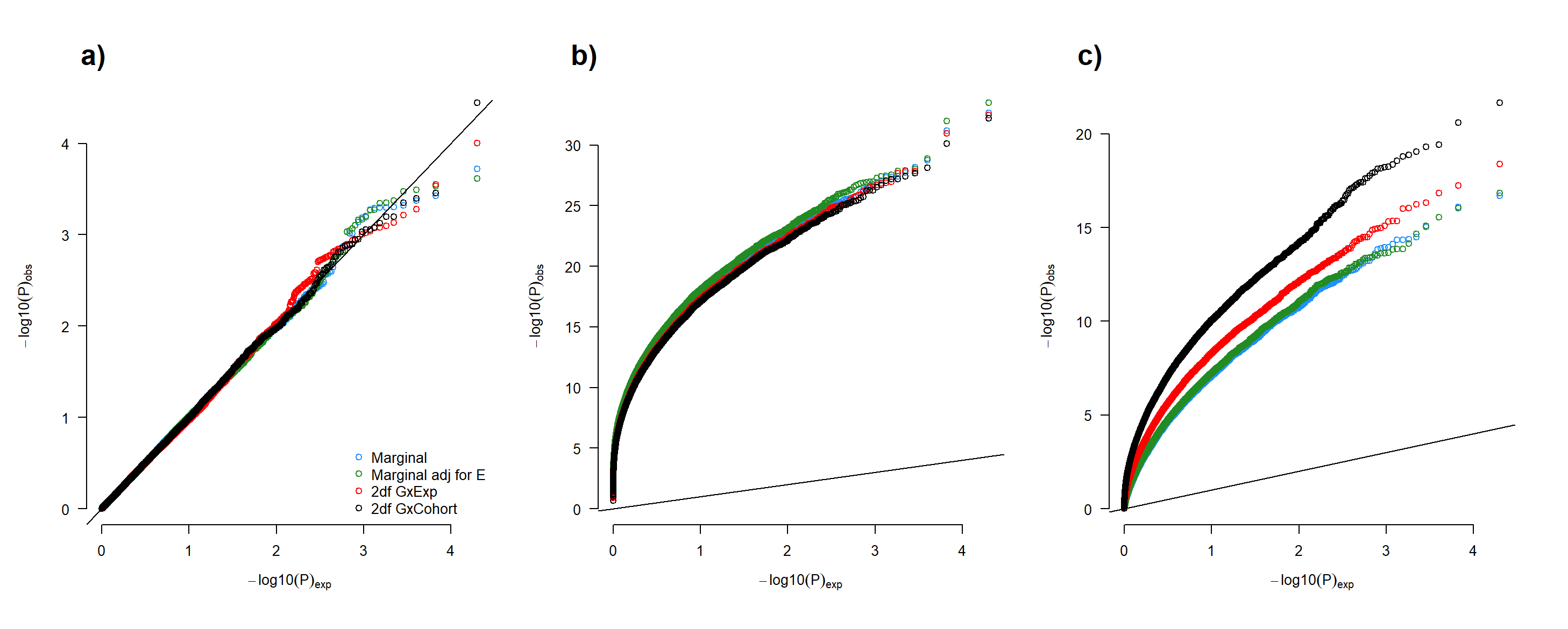


# References

1. Friedewald WT, Levy RI, Fredrickson DS. Estimation of the concentration of low-density lipoprotein cholesterol in plasma, without use of the preparative ultracentrifuge. Clin Chem. 1972;18(6):499-502.

2. Rao DC, Sung YJ, Winkler TW, Schwander K, Borecki I, Cupples LA, et al. Multiancestry Study of Gene-Lifestyle Interactions for Cardiovascular Traits in 610 475 Individuals From 124 Cohorts: Design and Rationale. Circulation Cardiovascular genetics. 2017;10(3).

3. de Vries PS, Brown MR, Bentley AR, Sung YJ, Winkler TW, Ntalla I, et al. Multi-Ancestry Genome-Wide Association Study of Lipid Levels Incorporating Gene-Alcohol Interactions. Am J Epidemiol. 2019.

4. Feitosa MF, Kraja AT, Chasman DI, Sung YJ, Winkler TW, Ntalla I, et al. Novel genetic associations for blood pressure identified via gene-alcohol interaction in up to 570K individuals across multiple ancestries. PloS one. 2018;13(6):e0198166.

5. Sung YJ, Winkler TW, de Las Fuentes L, Bentley AR, Brown MR, Kraja AT, et al. A Large-Scale Multi-ancestry Genome-wide Study Accounting for Smoking Behavior Identifies Multiple Significant Loci for Blood Pressure. American journal of human genetics. 2018;102(3):375-400.

6. Sung YJ, de Las Fuentes L, Winkler TW, Chasman DI, Bentley AR, Kraja AT, et al. A multi-ancestry genome-wide study incorporating gene-smoking interactions identifies multiple new loci for pulse pressure and mean arterial pressure. Hum Mol Genet. 2019.

7. Bentley AR, Sung YJ, Brown MR, Winkler TW, Kraja AT, Ntalla I, et al. Multi-ancestry genome-wide gene-smoking interaction study of 387,272 individuals identifies new loci associated with serum lipids. Nat Genet. 2019;51(4):636-48.

8. Manning AK, LaValley M, Liu CT, Rice K, An P, Liu Y, et al. Meta-analysis of gene-environment interaction: joint estimation of SNP and SNP x environment regression coefficients. Genet Epidemiol. 2011;35(1):11-8.

9. Willer CJ, Li Y, Abecasis GR. METAL: fast and efficient meta-analysis of genomewide association scans. Bioinformatics. 2010;26(17):2190-1.

10. Laville V, Majarian T, de Vries PS, Bentley AR, Feitosa MF, Sung YJ, et al. Deriving stratified effects from joint models investigating Gene-Environment Interactions. bioRxiv. 2019:693218.

11. Aschard H. A perspective on interaction effects in genetic association studies. Genet Epidemiol. 2016;40(8):678-88.

12. Laville V, Bentley AR, Prive F, Zhu X, Gauderman J, Winkler TW, et al. VarExp: estimating variance explained by genome-wide GxE summary statistics. Bioinformatics. 2018;34(19):3412-4.

13. Bulik-Sullivan BK, Loh PR, Finucane HK, Ripke S, Yang J, Schizophrenia Working Group of the Psychiatric Genomics C, et al. LD Score regression distinguishes confounding from polygenicity in genome-wide association studies. Nat Genet. 2015;47(3):291-5.

14. Finucane HK, Bulik-Sullivan B, Gusev A, Trynka G, Reshef Y, Loh PR, et al. Partitioning heritability by functional annotation using genome-wide association summary statistics. Nat Genet. 2015;47(11):1228-35.

15. Lu Q, Powles RL, Abdallah S, Ou D, Wang Q, Hu Y, et al. Systematic tissue-specific functional annotation of the human genome highlights immune-related DNA elements for late-onset Alzheimer's disease. PLoS Genet. 2017;13(7):e1006933.

16. Finucane HK, Reshef YA, Anttila V, Slowikowski K, Gusev A, Byrnes A, et al. Heritability enrichment of specifically expressed genes identifies disease-relevant tissues and cell types. Nat Genet. 2018;50(4):621-9.

17. Aschard H, Hancock DB, London SJ, Kraft P. Genome-wide meta-analysis of joint tests for genetic and gene-environment interaction effects. Hum Hered. 2010;70(4):292-300.

18. Dai JY, Kooperberg C, Leblanc M, Prentice RL. Two-stage testing procedures with independent filtering for genome-wide gene-environment interaction. Biometrika. 2012;99(4):929-44.

19. Genomes Project C, Auton A, Brooks LD, Durbin RM, Garrison EP, Kang HM, et al. A global reference for human genetic variation. Nature. 2015;526(7571):68-74.

20. Murcray CE, Lewinger JP, Conti DV, Thomas DC, Gauderman WJ. Sample size requirements to detect gene-environment interactions in genome-wide association studies. Genet Epidemiol. 2011;35(3):201-10.
